# CDK6-PI3K signaling axis is an efficient target for attenuating ABCB1/P-gp mediated multi-drug resistance (MDR) in cancer cells

**DOI:** 10.1186/s12943-022-01524-w

**Published:** 2022-04-22

**Authors:** Lei Zhang, Yidong Li, Chaohua Hu, Yangmin Chen, Zhuo Chen, Zhe-Sheng Chen, Jian-Ye Zhang, Shuo Fang

**Affiliations:** 1 State Key Laboratory of Structural Chemistry, Fujian Institute of Research on the Structure of Matter, Chinese Academy of Sciences, Fuzhou, 350002 China; 2 College of Pharmacy and Health Sciences, St. John’s University, Queens, New York, 11439 USA; 3 University of Chinese Academy of Sciences, Beijing, 100049 China; 4 National Engineering Research Center for Sugarcane, Fujian Agriculture and Forestry University, Fuzhou, 350002 China; 5 Key Laboratory of Molecular Target & Clinical Pharmacology and the State & NMPA Key Laboratory of Respiratory Disease, School of Pharmaceutical Sciences & the Fifth Affiliated Hospital, Guangzhou Medical University, Guangzhou, 511436 China; 6 The department of clinical oncology, Guangdong Provincial Key Laboratory of Digestive Cancer Research, Precision Medicine Center, The Seventh Affiliated Hospital, Sun Yat-Sen University, Shenzhen, 518107 China

**Keywords:** Multidrug resistance (MDR), cancer, Transcriptome sequencing, Cyclin dependent kinase 6 (CDK6), PI3K 110α/110β, Signaling axis, ATP-binding cassette (ABC) transporter ABCB1/P-gp

## Abstract

**Background:**

Multidrug resistance (MDR) mediated by ATP binding cassette subfamily B member 1 (ABCB1/P-gp) is a major cause of cancer chemotherapy failure, but the regulation mechanisms are largely unknown.

**Methods:**

Based on single gene knockout, we studied the regulation of CDK6-PI3K axis on ABCB1-mediated MDR in human cancer cells. CRISPR/Cas9 technique was performed in KB-C2 cells to knockout *cdk6* or *cdk4* gene*.* Western blot, RT-PCR and transcriptome analysis were performed to investigate target gene deletion and expression of critical signaling factors. The effect of *cdk4* or cdk6 deficiency on cell apoptosis and the cell cycle was analyzed using flow cytometry. In vivo studies were performed to study the sensitivity of KB-C2 tumors to doxorubicin, tumor growth and metastasis.

**Results:**

Deficiency of *cdk6* led to remarkable downregulation of ABCB1 expression and reversal of ABCB1-mediated MDR. Transcriptomic analysis revealed that CDK6 knockout regulated a series of signaling factors, among them, PI3K 110α and 110β, KRAS and MAPK10 were downregulated, and FOS-promoting cell autophagy and CXCL1-regulating multiple factors were upregulated. Notably, PI3K 110α/110β deficiency in-return downregulated CDK6 and the CDK6-PI3K axis synergizes in regulating ABCB1 expression, which strengthened the regulation of ABCB1 over single regulation by either CDK6 or PI3K 110α/110β. High frequency of alternative splicing (AS) of premature ABCB1 mRNA induced by CDK6, CDK4 or PI3K 110α/110β level change was confirmed to alter the ABCB1 level, among them 10 common skipped exon (SE) events were found. In vivo experiments demonstrated that loss of *cdk6* remarkably increased the sensitivity of KB-C2 tumors to doxorubicin by increasing drug accumulation of the tumors, resulting in remarkable inhibition of tumor growth and metastasis, as well as KB-C2 survival in the nude mice.

**Conclusions:**

CDK6-PI3K as a new target signaling axis to reverse ABCB1-mediated MDR is reported for the first time in cancers. Pathways leading to inhibition of cancer cell proliferation were revealed to be accompanied by CDK6 deficiency.

**Supplementary Information:**

The online version contains supplementary material available at 10.1186/s12943-022-01524-w.

## Introduction

Multidrug resistance (MDR) can develop in cancer cells that survive during chemotherapy. P-glycoprotein (P-gp), revealed as ATP-binding cassette (ABC) subfamily B member 1 (ABCB1) is an ABC transporter and its overexpression by various cancers can produce resistance to various chemotherapeutic drugs that have distinct structures and differing mechanisms of action [1], which hurdles the efficiency of chemotherapy against cancers. Despite the reversal efficacy of various types of ABCB1 inhibitors, mechanisms of inducing and regulating ABCB1-mediated MDR are rarely reported on the level of signaling pathways. Lack of understanding of ABCB1 regulation may lead to increased risk of side effects when treated with inhibitors, most of which are due to the lack of specificity.

An ideal target can exert its efficiency to inhibit cancer cell proliferation when it is used for attenuating MDR in cancer. Through CRISPR/Cas9 gene editing, we recently reported that 110α and 110β subunits of the phosphoinositide 3-kinase (PI3K) signaling pathway are potential targets for reversal of ABCB1/P-gp- and ABCG2/BCRP-mediated cancer MDR in a manner independent of AKT expression [2]. With the aim to characterize other efficient and safe targets that can be used to reverse ABCB1-mediated MDR in cancers, we further investigated the signaling pathways involved in the PI3K110α and 110β regulated ABCB1 expression.

The cyclin-dependent kinases (CDKs) are members of a family of serine-threonine kinases that regulate the cell cycle by altering cell proliferation and apoptosis [3–12]. Although many functional studies were based on the inhibitor-triggered variation of protein expression, which were frequently influenced by the cytotoxicity or non-specificity of the inhibitors instead of inhibition of CDKs themselves, some functions are demonstrated by protein-protein interactions. CDKs comprise three domains: the ATP binding and catalytic domain, the cyclin-binding domain, and the P13suc1 binding domain, in which P13suc1 can inhibit the activity of CDK and cell phase alteration [13–15]. By mediating a common kinase domain containing peptide, PSTAIRE, CDKs bind to their corresponding cyclin, forming a cyclin-CDK complex, where CDKs catalyze the phosphorylation of certain serine and threonine residues in target proteins. This regulates gene transcription and cell division, further promoting alteration of cell cycle between different phases, e.g., phase G1 to S, G2 to M, and exit from phase M [16].

CDK4 and CDK6 are essential kinases and are drawing increasing attention because they can drive cell proliferation by combining with cyclin D1, D2, and D3 [17]. Imbalance in the activity of CDK4 and CDK6 can induce dysregulation and uncontrolled cell division, which is a hallmark of cancers [17]. The interaction of CDK4 and CDK6 with D-type cyclins constitutively phosphorylates and inactivates the retinoblastoma protein (Rb), a tumor suppressor, causing the release of its binding partner, E2 transcription factor (E2F), allowing cancer cells to overcome pRb-dependent growth suppression [18]. Furthermore, CDK4 and CDK6 can bind to the proteins p15, p16, p21 and p27 [19, 20], and are regulated by cytosolic sialidase (Neu2) [21], FAT1 [22], SRY homology box 2 (SOX2) [23], reactive oxygen species (ROS) [24], and more. In addition, CDK6 may regulate epithelial-mesenchymal transition (EMT) via the CCNA2/MET pathway [25]. CDK6 also regulates malignant stem cell quiescence and facilitates nuclear factor kappaB (NF-κB) signaling [26]. The downregulation or inhibition of CDK6 or CDK 4 induces cellular apoptosis, suppresses tumor proliferation, migration, and invasion [27, 28].

Currently, whether CDK4 and/or CDK6 have direct regulatory effects on ABCB1 and ABCB1-mediated MDR in cancer, their roles, and mechanisms are unknown. Most of the inhibitors lack specificity, and some of them cannot accumulate inside the cells efficiently because they may be substrates of ABCB1 [29–31]. In this study, by gene knockout with CRISPR/Cas9 gene editing technique, we determined the regulation of CDK4 or CDK6 on ABCB1-mediated MDR in human epidermoid carcinoma MDR cell line KB-C2 which overexpresses ABCB1 and H460/MX80 which expresses ABCB1 at low level. Then we focused on the coordination of CDK6 with PI3K 110α/β to regulate ABCB1 expression, and their function to induce alternative splicing (AS) of *ABCB1* pre-mRNA. Importantly, this study demonstrated that CDK6 is a novel multi-functional target that may act as an on-off switch for ABCB1 expression, potentially reversing ABCB1-mediated MDR in cancers, and therefore, has significance in combination with cancer chemotherapy.

## Materials and methods

### Cells, plasmids, and chemicals

The human epidermoid carcinoma MDR cell line KB-C2 created by exposing KB-3-1 cells to increasing concentrations of colchicine and its parental cell line KB-3-1, which were used as a pair of models for studying ABCB1-mediated MDR in cancer [32], were kindly provided by Dr. Shin-ichi Akiyama (Kagoshima University, Kagoshima, Japan). Non-small cell lung cancer (NSCLC) NCl-H460 cell line was kindly provided by Drs. Susan E. Bates and Robert W. Robey (NIH, Bethesda, MD). H460/MX80 cells with enhanced drug-resistant ability were generated in our previous study [2]. KB-C2-k.o.110α, KB-C2-k.o.110β, MX80-k.o.110α, or MX80-k.o.110β gene-deficient cell populations were constructed using CRISPR/Cas9 editing technology according to our previous study [2]. The cells were cultivated with DMEM supplemented with 10% FBS and 1% penicillin/streptomycin in a humidified incubator containing 5% CO_2_ at 37 °C. CRISPR/Cas9 all-in-one plasmids, encoding single guide RNA (SgRNA) and Cas9, were purchased from GeneCopoeia Inc. (Rockville, MD).

Ribociclib was kindly provided by ChemieTek (Indianapolis, IN). Paclitaxel, colchicine, and DOX were purchased form Sigma Chemical Co. (St. Louis, MO). Mouse anti-ABCB1, HRP ligated or fluorescent secondary rabbit or goat-anti mouse antibodies, were purchased from Invitrogen, Thermo Fisher (Carlsbad, CA). Mouse-anti-CDK4 and CDK6 antibodies were purchased from R&D Systems (Minneapolis, MN). Rabbit-anti-CKAP4 antibody and Cy3- or FITC-labeled goat-anti-rabbit antibodies were purchased from Servicebio technology co., LTD (Wuhan, China). The other reagents were purchased from VWR International (West Chester, PA).

### Determination of cell viability: MTT assay

Exponentially growing cells were seeded into 96 well plates at 5 × 10^3^ cells/well. After 72 h of incubation, 20 μL of MTT (5 mg/mL) was added to each well. After incubation for an additional 4 h, the medium containing MTT was discarded and replaced with 150 μL of DMSO to dissolve the dark blue-purple crystal. The absorbance was measured at a wavelength of 490 nm, using an ELx 800 Universal Microplate Reader (Bio-Tek, Inc. Winooski, VT). The relative survival rate for the cells was analyzed using the SPSS 20 program (SPSS Inc., Chicago, IL) and the survival rate – drug concentration curves were generated using Origin 9.0 software (OriginLab Corporation, Northampton, MA). The concentration of drug required to inhibit cell viability by 50% (IC_50_ value) was determined using Origin 9.0 software.

### Western blot assay and immunofluorescence (IF) analysis

Parental KB-3-1 cells and MDR KB-C2 cells were incubated with 9 μM of ribociclib for 2 h and co-cultured with paclitaxel or colchicine for 24 to 72 h. For the Western blot assay, the cells were lysed with SDS lysate reagent, separated on a gradient polyacrylamide gel and transferred to a PVDF membrane. After blocking with 5% milk and washed with TBST buffer, the membrane was incubated with mouse anti-ABCB1 antibody at 4 °C for 2 h, adequately washed with TBST, and incubated with goat anti-mouse IgG-HRP at RT for 2 h. The membrane was then washed with TBST and exposed to the SignalFire™ ECL Reagent developing reagent (Cell Singling Technology, Danvers, MA), and the results were quantified using a AI600 RGB GEL Imaging System (GE, Fairfield, CT) set for the chemiluminescence mode.

For the IF analysis, KB-C2 and KB-3-1 cells were washed with PBS buffer (pH 8.0), fixed with 4% formaldehyde, followed by incubation with 0.1% of Triton-100 and then 6% BSA. The cells were co-incubated with ABCB1-specific antibodies (mouse originated, labeled with Alexa Fluor 488 (AF488); Santa Cruz Biotechnology, Inc. CA) for 1 h at 37 °C, washed with PBS for four times and stained with 0.5 μg mL^− 1^ of 4′,6-diamidino-2-phenylindole (DAPI) in PBS. Fluorescence was determined using a with Cytell™ Image Cytometer (GE Healthcare, Washington).

### Analysis of regulation of CDK6-PI3K axis on ABCB1 mediated drug resistance

CRISPR/Cas9 technique was performed in KB-C2 cells to knockout *cdk6* or *cdk4* gene*.* Western blot, RT-PCR and transcriptome analysis were performed to investigate target gene deletion and expression of critical signaling factors. Detailed procedure and other methods were described in supplemental materials and methods.

### CRISPR/Cas9 knockout and characterization for target gene deficiency

The cells were seeded into 48-well plates with serum-free DMEM. A polymeric GenePORTER transfection reagent was mixed with sgRNA/Cas9 all-in-one expression plasmid HCP254656-CG12–1-10 or HCP288574-CG12–1-10 to target AGTGTGAGAGTCCCCAATGG in *cdk4* (NM_000075.3) and GAAGAACGGAGGCCGTTTCG in *cdk6* (NM_001259.7), respectively (GeneCopoeia Inc., Rockville, MD). After incubation at room temperature for 10 min, the mixture was added to the cells. After incubation for 4 h, FBS was added to the cultures at 20% of the final volume ratio. To achieve a high transfection ratio, the same well of cells were transfected again after 48 h of cultivation. The drugs used in this study were not used during transfection and cell stabilization, and RT-PCR and Western blotting were performed to determine the transcription and expression of the target genes after stabilizing the gene deficient cells for 1 month via continuous cell cultivation.

To determine the transcripts for *cdk4*, primers (5′) GCTACCTCTCGATATG (3′) and (5′) AGCCTGGTGGGGGTGC (3′) were used for RT-PCR and for *cdk6*, the primers (5′) GAGAAGGACGGCCTGT (3′) and (5′) TCAAGTCTTGATCGAC (3′) were used.

### Analysis of CDK4 or CDK6 to regulate ABCB1 expression and ABCB1-mediated anti-drug efficacy

In the KB-C2 cells, either the *cdk4* or *cdk6* gene was deleted and the cells were cultured for 48 h and lysed with SDS lysate reagent. Western blot analysis of cell lysate was performed as described above for the determination of the effect of *cdk4* or *cdk6* knockout on the expression of the ABCB1 protein. The gene deficient cells were cultured with gradient concentrations (0.001, 0.01, 0.1, 1, 10 or 100 μM) of either colchicine or paclitaxel for 72 h. The MTT assay and IC_50_ determination was carried out as described above.

### Differential expression analysis and identification of AS events

Cells were lysed and Total RNA was extracted using the TRIzol® Reagent, according to the manufacturer’s instructions (Invitrogen). Genomic DNA was then removed using Dnase I (Takara Bio, Otsu, Japan). RNA quality was determined using a 2100 Bioanalyser (Agilent Technologies, Palo Alto, CA, USA) and quantified using ND-2000 (NanoDrop Technologies, Inc. Wilmington, DE, USA). RNA samples (5 μg) were used to construct the RNA-seq transcriptome library. The messenger RNA (mRNA) was fragmentated using a fragmentation buffer and isolated by the polyA selection method using oligo (dT) beads. According to Illumina’s library construction protocol, cDNA originated double-strands DNA (dscDNA) were synthesized, subjected to end-repair, phosphorylation and ‘A’ base addition, selected for cDNA target fragments of 200–300 bp, followed by PCR amplification using Phusion DNA polymerase (NEB) for 15 PCR cycles, and then sequenced using the Illumina HiSeq 4000 (2 × 150 bp read length). After map reading [33], differential expression genes (DEG) between two different samples were identified according to the fragments per kilobase of exon per million mapped reads (FRKM) method [34]. Network of gene clusters and protein-protein interaction were analyzed on metascape (http://metascape.org) [35] or STRING (http://string-db.org) platform, choosing *Homo Sapiens* as the control species. The data was visualized by drawing heatmaps using TBtools (https://github.com/CJ-Chen/TBtools). For the identification of AS events***.*** All of the AS events that occurred in the samples were identified by using the program, Multivariate Analysis of Transcript Splicing (MATS, http://rnaseq-mats.sourceforge.net/) [36].

### Analysis of cell apoptosis using flow cytometry

The KB-C2 cells and the derivative *cdk4-* or *cdk6-*deficient cells were seeded into a 24-well plate at 1 × 10^5^ cells per mL of medium and cultured with colchicine (1.25 μM) for 24 h. The cells were treated with 0.25% trypsin, followed by DMEM to terminate trypsin activity and washed two times with cold PBS. The cells incubated with the medium were used as a control group. Cell apoptosis was determined using an Annexin V-FITC Apoptosis Detection Kit (Solarbio Science & Technology Co., Ltd., Beijing, China), following the manufacturer’s instructions. The cellular apoptosis and necrosis ratios were analyzed using a CytoFLEX flow cytometer (Beckman Coulter, Brea, CA).

### Determining the effect of *cdk4* or *cdk6* deficiency on the cell cycle

The KB-C2 cells and *cdk4-* or *cdk6*-deficient cells derived from KB-C2 were seeded into a 24-well plate at 1 × 10^5^ cells per mL of medium and cultured with colchicine (1.25 μM) for 24 h. Next, the cells were treated with 0.25% trypsin, followed by DMEM and washed two times with PBS. The cells incubated with medium were used as a control group. The stage of the cell cycle was determined using a Cell Cycle Staining Kit (Multi Sciences (LianKe) Biotech, Co., Ltd., Hangzhou, China), following the manufacturer’s instructions. The cell populations were analyzed using a CytoFLEX flow cytometer (Beckman Coulter, Brea, CA) and the population range corresponding to the untreated KB-C2 cells in the best status (approximately 65% among all cell events), was selected as the gate for all the tested cell groups. All experiments were repeated in triplicate.

### In vivo studies

We established two tumor-bearing mouse models to evaluate the reversal of MDR tumor effect of *cdk6* deficiency in vivo. To establish the KB-C2 and KB-C2-k.o.cdk6 tumor-bearing mouse models, the aliquots (0.1 mL) of cancer cells in sterilized saline solution (1.0 × 10^7^ cells/mL) were subcutaneously inoculated into the back neck of the nude mice (BALB/c-nu). The mice were randomly divided into 4 groups (9 mice per group) with equivalent average starting tumor size (~ 50 mm^3^) and body weight (~ 23 g) treated with 0.9% saline (set as control) or DOX (2 mg Kg^− 1^), via the intraperitoneal route of administration for each tumor-bearing mouse model. Animals were examined daily, and their body weights and tumor sizes were determined using the formula: Tumor size = Length×Width×Height×π/6 [37]. The increased tumor sizes were calculated by subtracting the tumor sizes in the initial group from that of saline- or DOX-treated group at the subsequent measurement time. Animals were sacrificed 19 days after treatment.

The pathological morphology of liver, lung, spleen, heart, stomach and kidney of nude mice after drug withdrawal was analyzed by HE staining. Based on transcriptome analysis, genes with high level of expression in both KB-C2 and KB-C2-k.o.cdk6 are screened. Among them, cytoskeleton-associated protein 4 (CKAP4) was used as a biomarker for determination of distribution of KB-C2 or KB-C2-k.o.cdk6 in different organs including liver, lung and spleen. Immunofluorescence staining was then performed to analyze tumor cell migration in each group of tumor-bearing nude mice.

### Statistical analysis

All experiments were performed three times and the results were analyzed using Student’s unpaired *t*-test using SPSS 20.0 software (SPSS Inc., Chicago, IL). Data were expressed as the mean ± standard error of the mean (SEM). Results showing *P* values less than 0.05 were considered statistically significant.

### Experimental procedures

Reversal of MDR in cancer cells was studied by MTT assay. The expression level of ABCB1 was analyzed by Western blot assay and immunofluorescence (IF). the transcription and expression of the target genes were determined by RT-PCR and Western blotting, respectively, after stabilizing the gene deficient cells for 1 month via continuous cell cultivation. Up- or down- regulation of relative proteins and signaling pathways was studied by differential expression analysis and the mechanism was explored by identification of AS events and protein-protein interaction (PPI). Cell apoptosis and the cell cycle were analyzed by flow cytometry.

## Results

### Ribociclib, an inhibitor for CDK4/6, could downregulate ABCB1 expression

**Fig. 1:**
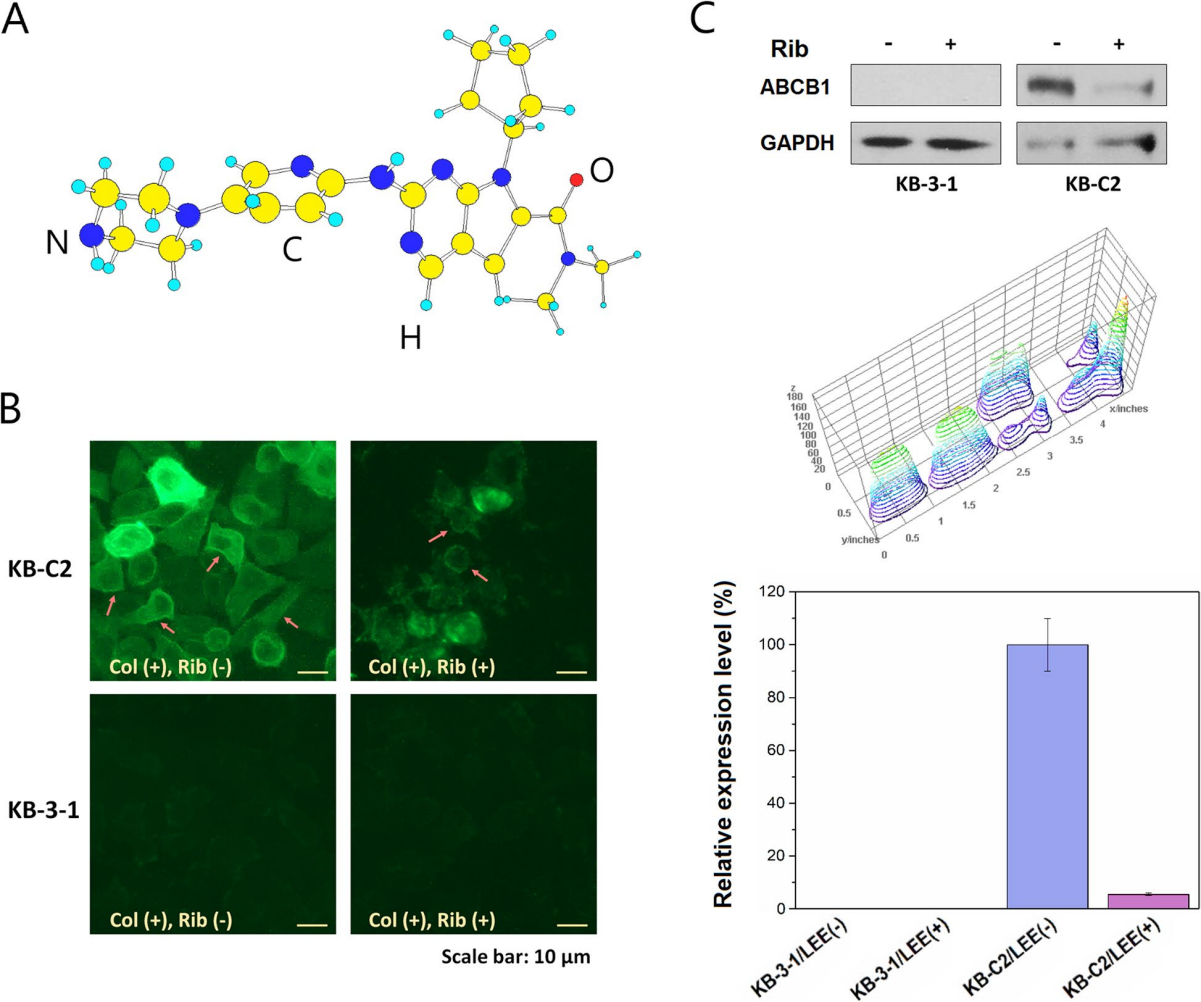
Downregulation of ABCB1 expression by ribociclib. **A** Molecular structure (3-dimentional) of ribociclib. **B**, **C** Immunofluorescence (IF) and Western blot data indicating ABCB1 expression was significantly downregulated by incubating the KB-C2 cells with 9 μM of ribociclib (Rib)/LEE011 for 72 h. Before the analysis, KB-C2 and KB-3-1 cells were incubated with 0.07 or 0.01 μM (lower than the IC_50_ values) of colchicine, respectively (to simulate the conditions with presence of colchicine during reversal of MDR and to maintain normal morphology in cells). ABCB1 expressed on the cell membrane is depicted by arrows in red

During screening the agents for reversal of ABCB1-mediated MDR, we found that ribociclib (LEE011), an inhibitor for CDK4/6, could significantly downregulate ABCB1 expression in MDR KB-C2 cells, as shown by immunofluorescence results and Western blot (Fig. 1). To our knowledge, most of the CDK4/6 inhibitors did not show the capability to reverse ABCB1-mediated MDR or downregulate ABCB1 expression. This could possibly be related to the low efficacy of CDK4 or CDK6 to regulate ABCB1 expression, or to the low intracellular accumulation efficiency and relatively poor specificity of many known inhibitors. Therefore, it is necessary to perform gene knockout to elucidate whether CDK4 or CDK6 deficiency has a role in reversing ABCB1-mediated drug resistance.

### The knockout of *cdk4* and *cdk6* genes using the CRISPR/Cas9 gene editing technique

**Fig. 2:**
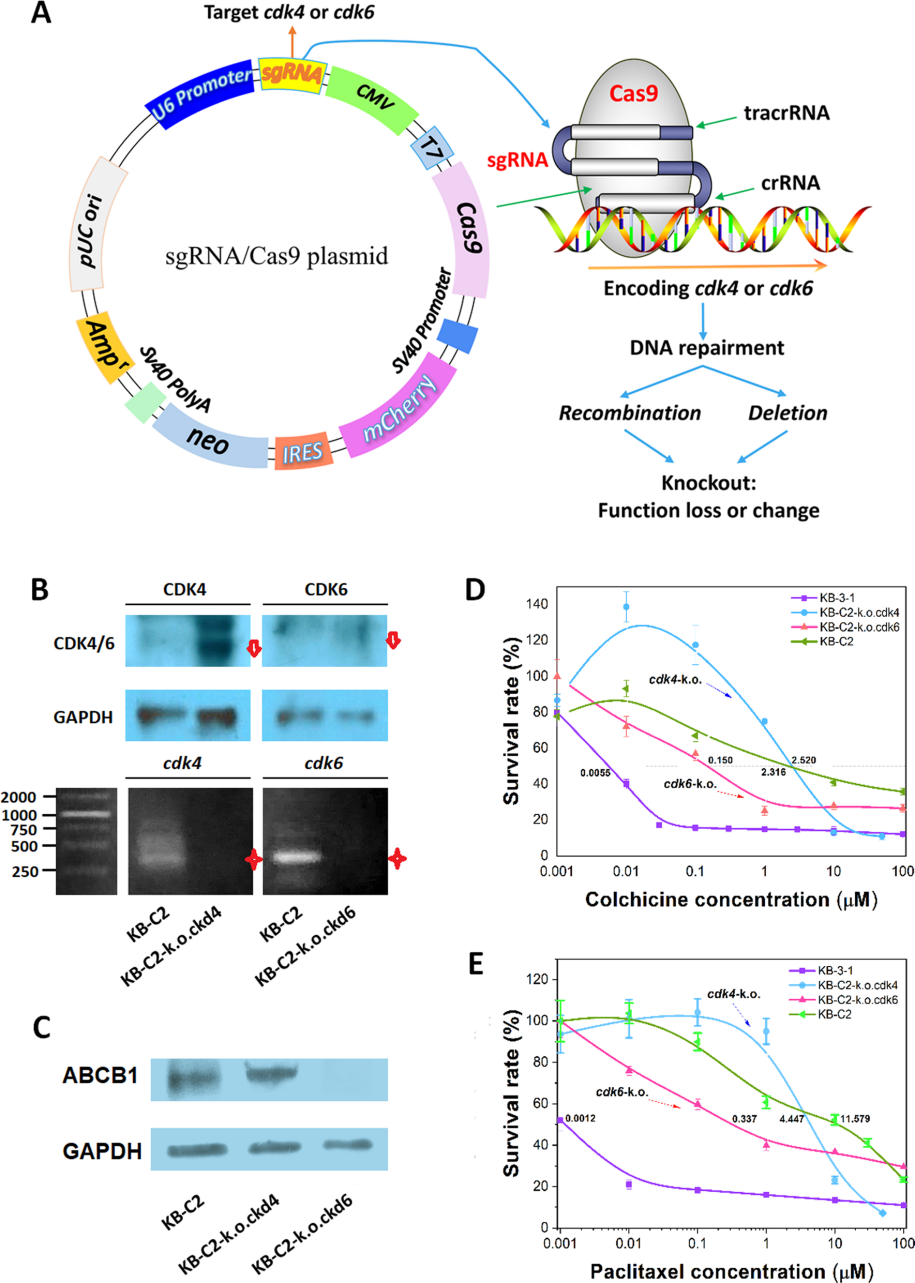
The effect of the deletion of *cdk4* or *cdk6* gene on the regulation of ABCB1 and the reversal of ABCB1-mediated MDR. **A** An all-in-one plasmid for CRISPR/Cas9 gene editing. **B** Western blot and RT-PCR analysis verifying the specific knockout of the targeted gene. The red stars depict the decrease in the copies of the target PCR. Truncated proteins are depicted by head-down arrows. **C** ABCB1 expression in the *cdk4*- or *cdk6*-deficient KB-C2 cells. The cell lysates containing identical amounts of total proteins were used for determining gene knockout in **(B)** and ABCB1 expression **(C)**. **D**, **E** The alteration of ABCB1-mediated MDR in the *cdk6-* or *cdk4-*deficient KB-C2 cells. Two substrate anticancer drugs of ABCB1, colchicine and paclitaxel, were used to compare ABCB1-mediated MDR in the cancer cells

To explore the potential mechanism of CDK4 or CDK6 in regulating ABCB1 expression, *cdk4* and *cdk6* genes were knocked out by transfecting KB-C2 cancer cells with the all-in-one CRISPR/Cas9 plasmid harboring the sgRNA that targets either the *cdk4* or *cdk6* genes (Fig. 2). The transfected cells were stabilized through continuous culture for at least 2 weeks. The deletions of *cdk4* or *cdk6* genes in KB-C2 produced a significant decrease in the expression of the CDK4 and CDK6 proteins, respectively (Fig. 2B). Western blot analysis (Fig. 2B), RT-PCR analysis (Fig. 2B) and transcriptome sequencing and quantification (Table S1) indicated that *cdk4* or *cdk6* gene were deleted by chromosomal recombination. The *cdk4* or *cdk6* deficient KB-C2 cells were named as KB-C2-k.o.cdk4 or KB-C2-k.o.cdk6, respectively.

### CDK6 is a potential target for on-off regulation of ABCB1 expression and ABCB1-mediated MDR

Amazingly, the gene deletion experiments indicated that the *cdk4* and *cdk6* genes have different roles in regulating ABCB1 expression and ABCB1-mediated MDR in KB-C2 cells. Compared with the KB-C2 cells, the deletion of the *cdk6* alone induced significant downregulation in the expression of the ABCB1 transporter in the KB-C2-k.o.cdk6 cells (Fig. 2C). The Western blot indicated that wild-type ABCB1 was remarkably downregulated in KB-C2-k.o.cdk6 cells (Fig. 2C) whereas trace amounts of ABCB1 homologous protein with a higher molecular weight might exist. Due to the decreased ABCB1 transporter expression, *cdk6*-deficient KB-C2 cells achieved remarkable reversal of MDR, showing significant increase in sensitivity to ABCB1 substrates colchicine (Fig. 2D) and paclitaxel (Fig. 2E), demonstrating that CDK6 regulates cancer MDR mediated by ABCB1 specifically.

Similarly, ABCB1 positive protein signal in Western blot was upregulated in KB-C2-k.o.cdk4 population, in which *cdk4* gene was knocked out (Fig. 2C). In line with this phenomenon, MDR was increased in the KB-C2-k.o.cdk4 (*cdk4* deficient KB-C2) cells incubated with low-to-medium concentrations of colchicine (< 2.5 μM) or paclitaxel (< 4 μM). However, MDR was not enhanced at higher concentrations of these drugs, indicating that KB-C2 cells deficient in CDK4 were more susceptible when incubated with higher concentrations of the drugs. Thus, our results clearly indicate that CDK6 and CDK4 function as possible on-off switches that regulate the expression of the ABCB1-mediated MDR, although CDK4 is homologous to CDK6. Native CDK6 may have a role in maintaining ABCB1-mediated MDR of cancers.

### Determining potential pathway(s) for CDK6 to regulate ABCB1 expression

**Fig. 3:**
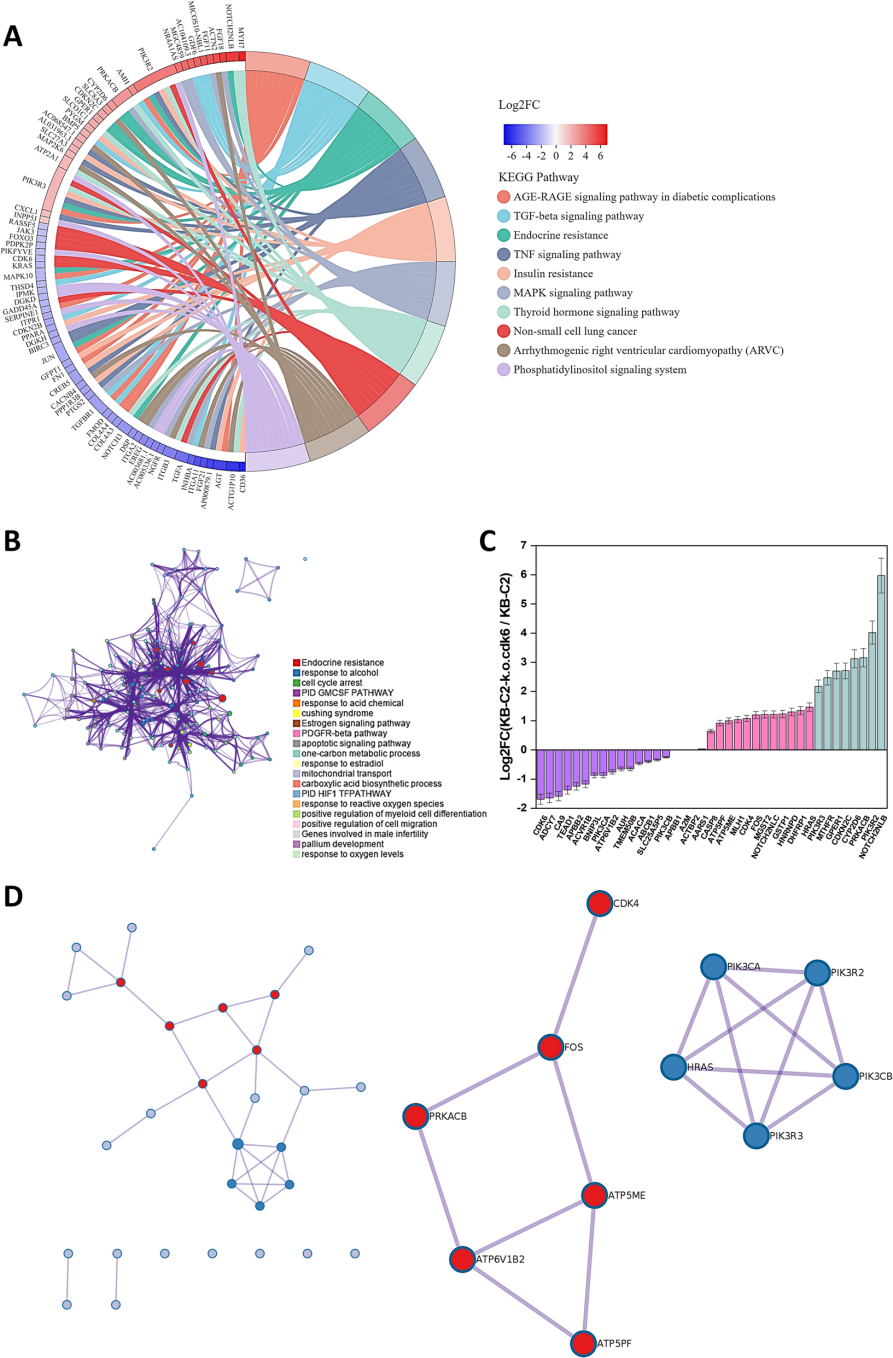
Category of the differential expression of genes and networks of the selected genes including the drug-resistant genes in KB-C2 and KB-C2-k.o.cdk6 cells. **A** Top10 KEGG pathways involved in the differential gene expression caused by knockout of *cdk6* in KB-C2. **B** Network of enriched genes. The items are colored by cluster ID, where nodes that share the same cluster ID are typically close to each other. **C** Differential degree of the drug-resistant and -selected genes in KB-C2-k.o.cdk6 cells. **D** Protein-protein interaction network of the proteins that are most correlated with acquired drug resistance to endocrine therapy of cancers

**Fig. 4:**
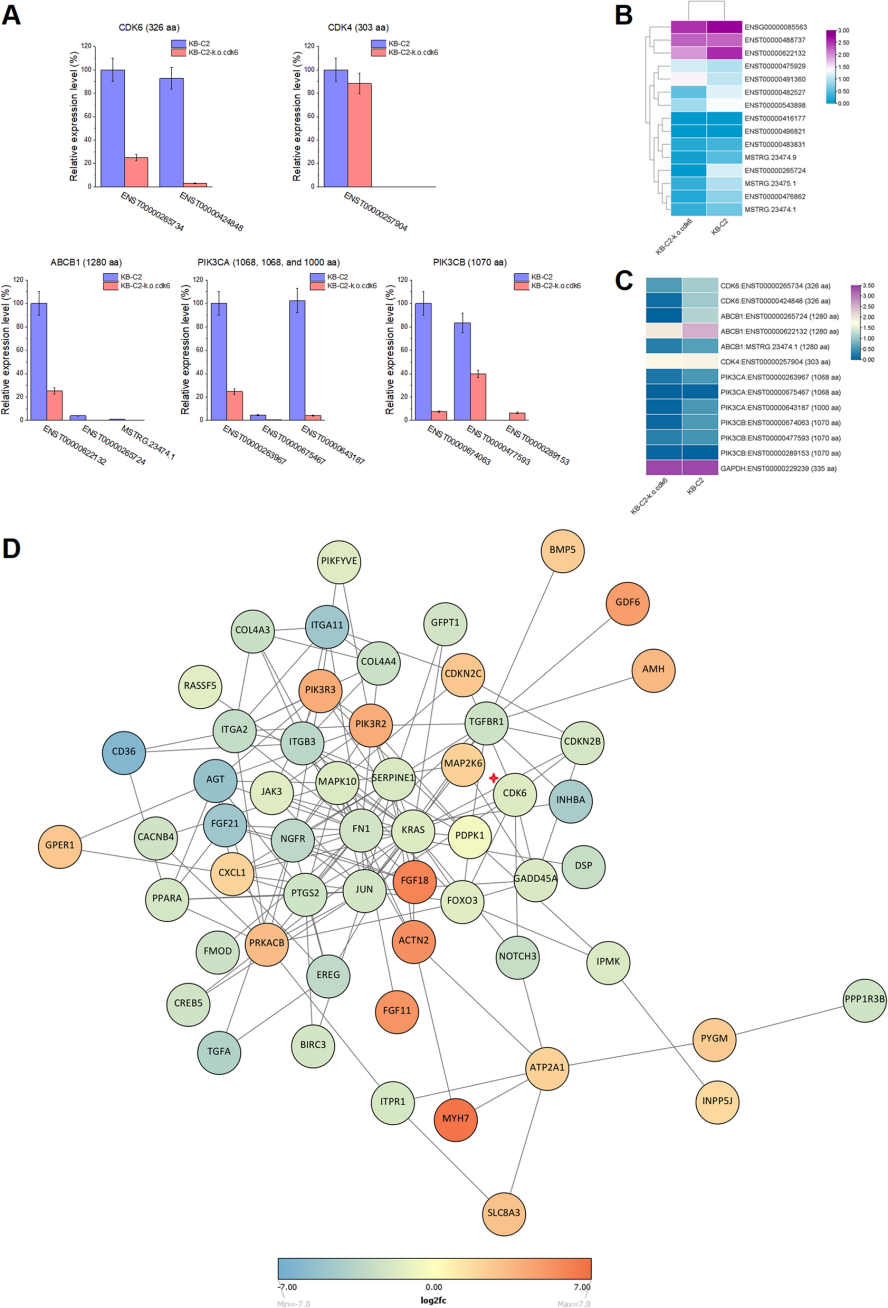
Cross-downregulation of CDK6-PI3K axis in the *cdk6*-deficient KB-C2 cells reduced the expression of ABCB1 by inducing responsive alternative splicing (AS) in the pre-mRNA of *ABCB1*. **A** Relative expression of the functional proteins including CDK6, CDK4, ABCB1, PIK3CA and PIK3CB in KB-C2 and the *cdk6*-deficient KB-C2 cell populations. Major transcripts according to each intact protein were calculated in the cells expressing identical level of GAPDH. **B** Heatmap showed that through AS of pre-mRNA in response to *cdk6* deletion, the level of the transcripts expressing functional ABCB1 protein was significantly downregulated whereas some transcripts expressing truncated inactive ABCB1 peptides (e.g., ENST00000488737) were upregulated in the KB-C2 cells with deleted *cdk6* gene. **C** Differential expression heatmap showing downregulation of PI3K 110α and 110β (encoded by *PIK3CA* and *PIK3CB* genes, respectively) in the KB-C2-k.o.cdk6 population as compared with the non-gene deficient KB-C2 cells. **D** Protein-protein interaction network showing the pathways linking the differently expressed proteins in the KB-C2 cells with deficiency of *cdk6.* This network reflects the factors that have most intimate relationships with CDK6, and the pathways that are regulated by deletion of *cdk6* and belong to different biological functions. Yellow represents the protein with no significant difference in expression. From green to blue, the downregulation of proteins is gradually obvious. From orange to red, the up regulation of proteins is gradually obvious

The downregulation of ABCB1 induced by CDK6 deficiency in KB-C2-k.o.cdk6 attracted our attention. In order to uncover the pathway for CDK6 to downregulate ABCB1 expression and reverse MDR, we analyzed differential gene expression based on the transcriptome sequencing database of the KB-C2-k.o.cdk6 cell population. Among the 8974 genes with significant variance in gene expression (p-adjust < 0.001, differential folds > 1.3), the Top10 KEGG pathways involved in the differential gene expression caused by knockout of CDK6 are intimately related to advanced glycation end product (AGE)-AGE receptor (AGE-RAGE) signaling, transforming growth factor-β signaling, endocrine resistance (acquired drug resistance to endocrine therapy of cancers), tumor necrosis factor signaling, et al., among which CDK6 is one of the top10 factors (Fig. 3A). Among the proteins most correlated with endocrine resistance, neurogenic locus notch homolog protein 3 (NOTCH3), transcription factor AP-1 (JUN), and mitogen-activated protein kinase 10 (MAPK10) were downregulated, whereas phosphatidylinositol 3-kinase regulatory subunit gamma (PIK3R3), G-protein coupled estrogen receptor 1 (GPER1), cyclin-dependent kinase inhibitor 2C (CDKN2C), cytochrome P450 2D6 (CYP2D6), cAMP-dependent protein kinase catalytic subunit beta (PRKACB), phosphatidylinositol 3-kinase regulatory subunit beta (PIK3R2) and notch 2 N-terminal like B (NOTCH2NLB) were upregulated. Interactions between 1) MAPK10 and JUN, 2) PIK3R2 and PIK3R3 were revealed (Fig. S1). We then selected the “drug resistance” associated genes from KEGG category. Network analysis showed that they belong to one or more groups associated with different types of resistance or response to drugs or stimuli (Fig. 3B). The differential expression of these genes in KB-C2 and KB-C2-k.o.cdk6 are shown in Fig. 3C. Protein-protein interaction (PPI) analysis revealed: 1) the signaling pathways involving CDK4, FOS, PRKACB, brain V-type proton ATPase subunit B2 (ATP6V1B2), mitochondrial ATP synthase subunit e (ATP5ME) and mitochondrial ATP synthase-coupling factor 6 (ATP5PF), and 2) PI3K/HRAS signaling pathways. However, no regulation between these genes and ABCB1 expression was retrieved, either using metascape or STRING platform (Fig. 3D, Fig. S2). Nevertheless, the experimental data showed that a series of endocrine resistance-related signaling factors were upregulated or downregulated in the *cdk6*-deficient cells. Among them, FOS-promoting cell autophagy [38] was upregulated, whereas PI3K (PIK3CA/PIK3CB), which is a key signaling factor in cancer and was found to regulate ABCB1-mediated MDR in our recent study [2], was downregulated accompanying the remarkable downregulation of ABCB1 (Fig. 4A, B and C).

### Cross-downregulation between CDK6 and PI3K 110α/110β suppresses ABCB1 expression in *cdk6* deficient cancer cells

Our previous study revealed that P110α and P110β could be potential targets that regulate ABCB1-mediated MDR in cancers [2]. It is of great interest whether *cdk6* gene downregulation may cause co-downregulation of PI3K 110α and 110β expression. In so doing, both P110α and P110β expression levels are significantly downregulated with the knockout of *cdk6* gene (Fig. 4A and C, Table S1). Therefore, downregulation of ABCB1 expression and reversal of ABCB1-mediated MDR in KB-C2-k.o.cdk6 could possibly, at least partially be induced by the downregulation of P110α and P110β resulted from knocking out the *cdk6* gene.

Furthermore, knockout of *cdk6*, *PIK3CA* or *PIK3CB* in KB-C2 indicated that CDK6 and P110α/P110β could positively regulate each other (Tables S1, S2). This reverse regulation of P110α/P110β on CDK6 was also found in H460/MX80 cells via knockout of *PIK3CA* or *PIK3CB* genes (Table S2), and further strengthened the possibility that cross regulation exists between CDK6 and P110α/P110β, which co-interact and synergize to regulate ABCB1 expression and ABCB1-mediated MDR in KB-C2 and H460/MX80 cells (Fig. S2).

### CDK6, CDK4 and PI3K 110α/110β regulate ABCB1 expression by inducing AS of pre-mRNA of ABCB1 gene

By using the TPM (transcripts per million mapped reads) level of GAPDH encoding transcript (ENST00000229239) as the standard, the levels of transcripts encoding native functional CDK6 (326 aa), i.e. ENST00000265734 and ENST00000424848, were dramatically decreased in KB-C2-k.o.cdk6 cell populations, and 25 and 3%, respectively, remained as compared with those detected in non-gene-deficient KB-C2 cells (Fig. 4A,C Table S1). The ABCB1 transcripts ENST00000622132 and ENST00000265724 encoding functional ABCB1 (1280 aa) protein decreased to 25 and 0%, respectively, in KB-C2-k.o.cdk6 as compared with KB-C2 MDR cells (Fig. 4B, Table S1).

To study the mechanisms of the altered ABCB1 expression induced by CDK6 or CDK4, we analyzed the AS of the pre-mature mRNA of ABCB1 gene, which expresses protein ABCB1. The results demonstrated that in either KB-C2 cell population with *cdk6* or *cdk4* deficiency, a high frequency of skipped exon (SE) splicing was detected in the *ABCB1* pre-mRNA (Table 1). Among the 14 SE events in *ABCB1* pre-mRNA of KB-C2-k.o.cdk6 cells, 12 of them were novel chromosomal AS events. Thirteen SE were found in KB-C2-k.o.cdk4 cells, where 10 were novel chromosomal AS. The high frequency of AS events in *ABCB1* pre-mRNA in response to *cdk6* or *cdk4* deficiency generated altered expression level of the ABCB1 protein, further demonstrating that CDK6 and CDK4 may regulate *ABCB1* expression by generating responsive AS in *ABCB1* pre-mRNA.

**Table 1:** Skipped exon (SE) evidence of *ABCB1* transcripts detected in transcriptomes from KB-C2-k.o.cdk6, KB-C2-k.o.cdk4, KB-C2-110α, KB-C2-110β as compared with KB-C2 transcriptome

**(I) KB-C2-k.o.cdk6 vs KB-C2**												
**AS ID**	**Gene ID**	**Gene name**	**Novel AS**	**Chr**	**Diff significant**	**P Value Junction Count Only**	**Exon Start (0 base)**	**Exon End**	**Upstream ES**	**Upstream EE**	**Downstream ES**	**Downstream EE**
SE_46658	ENSG00000085563	ABCB1	yes	7	no	1	87516508	87516665	87515227	87515428	87519325	87519466
SE_46659	ENSG00000085563	ABCB1	yes	7	no	1	87536457	87536541	87531293	87531497	87539267	87539345
SE_46657	ENSG00000085563	ABCB1	yes	7	no	1	87505896	87506043	87502690	87504449	87509274	87509481
SE_46669	ENSG00000085563	ABCB1	no	7	no	0.9038720596	87602959	87603140	87600812	87601078	87713160	87713295
SE_46668	ENSG00000085563	ABCB1	yes	7	yes	7.74577644824E-006	87601966	87603140	87600754	87601078	87605645	87605775
SE_46663	ENSG00000085563	ABCB1	yes	7	no	1	87550724	87550838	87550467	87550578	87553760	87553932
SE_46662	ENSG00000085563	ABCB1	yes	7	no	1	87550170	87550296	87549850	87550054	87550467	87550578
SE_46661	ENSG00000085563	ABCB1	yes	7	no	1	87549347	87549518	87545862	87546024	87549850	87550054
SE_46660	ENSG00000085563	ABCB1	yes	7	no	1	87541356	87541464	87539267	87539345	87544128	87544275
SE_46667	ENSG00000085563	ABCB1	yes	7	no	1	87595765	87595814	87585511	87585680	87600116	87600190
SE_46666	ENSG00000085563	ABCB1	yes	7	no	1	87595765	87595814	87505896	87506043	87600116	87600190
SE_46665	ENSG00000085563	ABCB1	yes	7	no	0.0682123258	87566784	87566976	87566069	87566241	87570171	87570223
SE_46664	ENSG00000085563	ABCB1	yes	7	no	0.4481719285	87566069	87566241	87561262	87561387	87570171	87570223
SE_46670	ENSG00000085563	ABCB1	no	7	no	0.7879487815	87605645	87605775	87600754	87601078	87713160	87713294
**(II) KB-C2-k.o.cdk4 vs KB-C2**												
**AS ID**	**Gene ID**	**Gene name**	**Novel AS**	**Chr**	**Diff significant**	**P Value Junction Count Only**	**Exon Start (0 base)**	**Exon End**	**Upstream ES**	**Upstream EE**	**Downstream ES**	**Downstream EE**
SE_47404	ENSG00000085563	ABCB1	yes	7	no	0.1867192198	87566784	87566976	87566069	87566241	87570171	87570223
SE_47405	ENSG00000085563	ABCB1	yes	7	no	1	87595765	87595814	87585511	87585680	87600116	87600190
SE_47406	ENSG00000085563	ABCB1	yes	7	no	0.4757658641	87601966	87603140	87600754	87601078	87605645	87605775
SE_47407	ENSG00000085563	ABCB1	no	7	no	0.1348013017	87602959	87603140	87600812	87601078	87713160	87713295
SE_47400	ENSG00000085563	ABCB1	yes	7	no	1	87549347	87549518	87545862	87546024	87549850	87550054
SE_47401	ENSG00000085563	ABCB1	yes	7	no	1	87550170	87550296	87549850	87550054	87550467	87550578
SE_47402	ENSG00000085563	ABCB1	yes	7	no	1	87550724	87550838	87550467	87550578	87553760	87553932
SE_47403	ENSG00000085563	ABCB1	yes	7	no	0.3242107276	87566069	87566241	87561262	87561387	87570171	87570223
SE_47408	ENSG00000085563	ABCB1	no	7	no	0.0695066227	87605645	87605775	87600754	87601078	87713160	87713294
SE_47409	ENSG00000085563	ABCB1	no	7	yes	2.4192194914E-009	87605645	87605775	87601966	87603140	87713160	87713294
SE_47397	ENSG00000085563	ABCB1	yes	7	no	1	87519325	87519466	87516508	87516665	87520775	87520876
SE_47398	ENSG00000085563	ABCB1	yes	7	no	1	87536457	87536541	87531293	87531497	87539267	87539345
SE_47399	ENSG00000085563	ABCB1	yes	7	no	1	87541356	87541464	87539267	87539345	87544128	87544275
**(III) KB-C2-k.o.110α vs KB-C2**												
**AS ID**	**Gene ID**	**Gene name**	**Novel AS**	**Chr**	**Diff significant**	**P Value Junction Count Only**	**Exon Start (0 base)**	**Exon End**	**Upstream ES**	**Upstream EE**	**Downstream ES**	**Downstream EE**
SE_50129	ENSG00000085563	ABCB1	no	7	no	0.1120059639	87605645	87605775	87601966	87603140	87713160	87715224
SE_50128	ENSG00000085563	ABCB1	no	7	yes	2.83848985472E-006	87605645	87605775	87600754	87601078	87713160	87715224
SE_50127	ENSG00000085563	ABCB1	no	7	yes	0	87602959	87603140	87600754	87601078	87713160	87713295
SE_50125	ENSG00000085563	ABCB1	no	7	no	0.0641212769	87601272	87601371	87600754	87601078	87602959	87603140
SE_50123	ENSG00000085563	ABCB1	yes	7	no	1	87595765	87595814	87585511	87585680	87600116	87600190
SE_50122	ENSG00000085563	ABCB1	yes	7	no	1	87570171	87570223	87561262	87561387	87585511	87585680
SE_50121	ENSG00000085563	ABCB1	yes	7	no	0.0842138339	87566784	87566976	87566069	87566241	87570171	87570223
SE_50112	ENSG00000085563	ABCB1	yes	7	no	1	87519325	87519466	87516508	87516665	87520775	87520876
SE_50116	ENSG00000085563	ABCB1	yes	7	no	1	87550170	87550296	87549850	87550054	87550467	87550578
SE_50117	ENSG00000085563	ABCB1	yes	7	no	1	87550724	87550838	87550467	87550578	87553760	87553932
SE_50114	ENSG00000085563	ABCB1	yes	7	no	0.5111213437	87541356	87541464	87539267	87539345	87544128	87544275
SE_50126	ENSG00000085563	ABCB1	yes	7	no	0.2905363226	87601966	87603140	87600754	87601078	87605645	87605775
SE_50124	ENSG00000085563	ABCB1	yes	7	no	0.2672547758	87601272	87601371	87600754	87601078	87601966	87603140
SE_50120	ENSG00000085563	ABCB1	yes	7	no	0.5376920457	87566784	87566976	87561262	87561387	87570171	87570223
SE_50113	ENSG00000085563	ABCB1	yes	7	no	1	87536457	87536541	87531293	87531497	87539267	87539345
SE_50115	ENSG00000085563	ABCB1	yes	7	no	1	87549347	87549518	87545862	87546024	87549850	87550054
SE_50118	ENSG00000085563	ABCB1	yes	7	no	1	87566069	87566241	87561262	87561387	87566784	87566976
SE_50119	ENSG00000085563	ABCB1	yes	7	no	0.4481965282	87566069	87566241	87561262	87561387	87570171	87570223
**(V) KB-C2-k.o.110β vs KB-C2**												
**AS ID**	**Gene ID**	**Gene name**	**Novel AS**	**Chr**	**Diff significant**	**P Value Junction Count Only**	**Exon Start (0 base)**	**Exon End**	**Upstream ES**	**Upstream EE**	**Downstream ES**	**Downstream EE**
SE_49198	ENSG00000085563	ABCB1	yes	7	no	0.4057523949	87601272	87601371	87600754	87601078	87601966	87603140
SE_49199	ENSG00000085563	ABCB1	no	7	yes	0.0006903622	87601272	87601371	87600754	87601078	87602959	87603140
SE_49190	ENSG00000085563	ABCB1	yes	7	no	1	87550170	87550296	87549850	87550054	87550467	87550578
SE_49191	ENSG00000085563	ABCB1	yes	7	no	0.365137306	87550467	87550578	87549850	87550054	87553760	87553932
SE_49192	ENSG00000085563	ABCB1	yes	7	no	1	87550724	87550838	87550467	87550578	87553760	87553932
SE_49193	ENSG00000085563	ABCB1	yes	7	no	1	87566069	87566241	87561262	87561387	87566784	87566976
SE_49194	ENSG00000085563	ABCB1	yes	7	no	0.448132556	87566069	87566241	87561262	87561387	87570171	87570223
SE_49195	ENSG00000085563	ABCB1	yes	7	no	0.5487589032	87566784	87566976	87561262	87561387	87570171	87570223
SE_49196	ENSG00000085563	ABCB1	no	7	no	0.9285527169	87566784	87566976	87566069	87566241	87570171	87570223
SE_49197	ENSG00000085563	ABCB1	yes	7	no	1	87595765	87595814	87585511	87585680	87600116	87600190
SE_49189	ENSG00000085563	ABCB1	yes	7	no	1	87549347	87549518	87545862	87546024	87549850	87550054
SE_49188	ENSG00000085563	ABCB1	yes	7	no	1	87541356	87541464	87539267	87539345	87544128	87544275
SE_49187	ENSG00000085563	ABCB1	yes	7	no	1	87536457	87536541	87531293	87531497	87539267	87539345
SE_49186	ENSG00000085563	ABCB1	yes	7	no	1	87516508	87516665	87515227	87515428	87519325	87519466
SE_49200	ENSG00000085563	ABCB1	no	7	yes	1.1990408666E-014	87601272	87601371	87600754	87601078	87713160	87715224
SE_49201	ENSG00000085563	ABCB1	yes	7	yes	0.000649673	87601966	87603140	87600754	87601078	87605645	87605775
SE_49202	ENSG00000085563	ABCB1	no	7	yes	0	87602959	87603140	87600754	87601078	87713160	87713295
SE_49203	ENSG00000085563	ABCB1	yes	7	yes	0.0067345237	87602959	87603140	87601272	87601371	87713160	87713295
SE_49204	ENSG00000085563	ABCB1	no	7	yes	0	87605645	87605775	87600754	87601078	87713160	87715224
SE_49205	ENSG00000085563	ABCB1	no	7	no	0.0538934718	87605645	87605775	87601966	87603140	87713160	87715224

**(I) KB-C2-k.o.cdk6 vs KB-C2**											
**IJC KB-C2**	**SJC KB-C2**	**IJC KB-C2-k.o.cdk6**	**SJC KB-C2-k.o.cdk6**	**Inc Form Len**	**Skip Form Len**	**Average Inc Level1 (average PSI 1)**	**Average Inc Level2 (average PSI 2)**	**Increase Inclusion KB-C2 (ΔPSI>0)**	**Increase Exclusion KB-C2 (ΔPSI<0)**	**Increase Inclusion KB-C2-k.o.cdk6 (ΔPSI<0)**	**Increase Exclusion KB-C2-k.o.cdk6 (ΔPSI>0)**
7907	9	5933	0	298	149	0.998	1	no	yes	yes	no
2821	1	2975	0	232	149	0.999	1	no	yes	yes	no
12259	1	7047	0	295	149	1	1	no_difference	no_difference	no_difference	no_difference
11	35	38	129	298	149	0.136	0.128	yes	no	no	yes
0	2	50	1	298	149	0	0.962	no	yes	yes	no
2116	2	4377	3	262	149	0.998	0.999	no	yes	yes	no
2845	2	4885	4	274	149	0.999	0.998	yes	no	no	yes
3787	9	5847	1	298	149	0.995	1	no	yes	yes	no
2626	0	3200	14	256	149	1	0.993	yes	no	no	yes
1170	0	3565	27	197	149	1	0.99	yes	no	no	yes
349	1	931	0	197	149	0.996	1	no	yes	yes	no
1491	45	4298	83	298	149	0.943	0.963	no	yes	yes	no
857	0	1995	14	298	149	1	0.986	yes	no	no	yes
2	35	4	129	278	149	0.03	0.016	yes	no	no	yes
**(II) KB-C2-k.o.cdk4 vs KB-C2**											
**IJC KB-C2**	**SJC KB-C2**	**IJC KB-C2-k.o.cdk4**	**SJC KB-C2-k.o.cdk4**	**Inc Form Len**	**Skip Form Len**	**Average Inc Level1 (average PSI 1)**	**Average Inc Level2 (average PSI 2)**	**Increase Inclusion KB-C2 (ΔPSI>0)**	**Increase Exclusion KB-C2 (ΔPSI<0)**	**Increase Inclusion KB-C2-k.o.cdk4 (ΔPSI<0)**	**Increase Exclusion KB-C2-k.o.cdk4 (ΔPSI>0)**
3892	56	4300	83	341	149	0.968	0.958	yes	no	no	yes
2933	7	3565	27	198	149	0.997	0.99	yes	no	no	yes
54	0	50	1	1323	149	1	0.849	yes	no	no	yes
46	101	38	129	330	149	0.171	0.117	yes	no	no	yes
5497	14	5847	1	320	149	0.995	1	no	yes	yes	no
5076	4	4885	4	275	149	0.999	0.998	yes	no	no	yes
4228	0	4377	3	263	149	1	0.999	yes	no	no	yes
1914	1	1995	14	321	149	0.999	0.985	yes	no	no	yes
9	101	4	129	279	149	0.045	0.016	yes	no	no	yes
20	0	3	11	279	149	1	0.127	yes	no	no	yes
5188	1	5459	0	290	149	1	1	no_difference	no_difference	no_difference	no_difference
3032	4	2975	0	233	149	0.998	1	no	yes	yes	no
3431	0	3200	14	257	149	1	0.993	yes	no	no	yes
**(III) KB-C2-k.o.110α vs KB-C2**											
**IJC KB-C2**	**SJC KB-C2**	**IJC KB-C2-k.o.110α**	**SJC KB-C2-k.o.110α**	**Inc Form Len**	**Skip Form Len**	**Average Inc Level1 (average PSI 1)**	**Average Inc Level2 (average PSI 2)**	**Increase Inclusion KB-C2 (ΔPSI>0)**	**Increase Exclusion KB-C2 (ΔPSI<0)**	**Increase Inclusion KB-C2-k.o.110α (ΔPSI<0)**	**Increase Exclusion KB-C2-k.o.110α (ΔPSI>0)**
11	14	3	11	278	149	0.296	0.128	yes	no	no	yes
3	0	4	129	278	149	1	0.016	yes	no	no	yes
30	0	38	129	298	149	1	0.128	yes	no	no	yes
38	16	30	27	247	149	0.589	0.401	yes	no	no	yes
4622	7	3565	27	197	149	0.998	0.99	yes	no	no	yes
818	1	701	0	200	149	0.998	1	no	yes	yes	no
5347	145	4298	83	298	149	0.949	0.963	no	yes	yes	no
7533	7	5459	0	289	149	0.998	1	no	yes	yes	no
6308	5	4885	4	274	149	0.999	0.998	yes	no	no	yes
5327	2	4377	3	262	149	0.999	0.999	no_difference	no_difference	no_difference	no_difference
4489	32	3200	14	256	149	0.988	0.993	no	yes	yes	no
58	0	50	1	298	149	1	0.962	yes	no	no	yes
66	50	47	50	247	149	0.443	0.362	yes	no	no	yes
2856	2	2099	14	298	149	0.999	0.987	yes	no	no	yes
4051	26	2975	0	232	149	0.99	1	no	yes	yes	no
8098	9	5847	1	298	149	0.998	1	no	yes	yes	no
4749	1	4111	0	298	149	1	1	no_difference	no_difference	no_difference	no_difference
2402	2	1995	14	298	149	0.998	0.986	yes	no	no	yes
**(V) KB-C2-k.o.110β vs KB-C2**											
**IJC KB-C2**	**SJC KB-C2**	**IJC KB-C2-k.o.110β**	**SJC KB-C2-k.o.110β**	**Inc Form Len**	**Skip Form Len**	**Average Inc Level1 (average PSI 1)**	**Average Inc Level2 (average PSI 2)**	**Increase Inclusion KB-C2 (ΔPSI>0)**	**Increase Exclusion KB-C2 (ΔPSI<0)**	**Increase Inclusion KB-C2-k.o.110β (ΔPSI<0)**	**Increase Exclusion KB-C2-k.o.110β (ΔPSI>0)**
21	30	47	50	247	149	0.297	0.362	no	yes	yes	no
15	53	30	27	247	149	0.146	0.401	no	yes	yes	no
6090	5	4885	4	274	149	0.998	0.998	no_difference	no_difference	no_difference	no_difference
12	1	5	0	259	149	0.873	1	no	yes	yes	no
5428	7	4377	3	262	149	0.998	0.999	no	yes	yes	no
4885	1	4111	0	298	149	1	1	no_difference	no_difference	no_difference	no_difference
2345	0	1995	14	298	149	1	0.986	yes	no	no	yes
2793	0	2099	14	298	149	1	0.987	yes	no	no	yes
5439	107	4298	83	298	149	0.962	0.963	no	yes	yes	no
4078	0	3565	27	197	149	1	0.99	yes	no	no	yes
7421	5	5847	1	298	149	0.999	1	no	yes	yes	no
4110	0	3200	14	256	149	1	0.993	yes	no	no	yes
3805	9	2975	0	232	149	0.996	1	no	yes	yes	no
8034	1	5933	0	298	149	1	1	no_difference	no_difference	no_difference	no_difference
19	0	28	129	247	149	1	0.116	yes	no	no	yes
53	12	50	1	298	149	0.688	0.962	no	yes	yes	no
109	0	38	129	298	149	1	0.128	yes	no	no	yes
58	6	15	0	298	149	0.829	1	no	yes	yes	no
33	0	4	129	278	149	1	0.016	yes	no	no	yes
44	56	3	11	278	149	0.296	0.128	yes	no	no	yes

The knockout of either *PIK3CA* (encoding P110α) or *PIK3CB* (encoding P110β) in KB-C2 cells also resulted in a number of novel AS events in *ABCB1* pre-mRNA and downregulation of ABCB1 protein expression (Table 1). Interestingly, 10 common SE positions in *ABCB1* were found in the four types of gene-deficient cell populations, i.e., KB-C2 cells deficient in *cdk6*, *cdk4*, *PIK3CA* and *PIK3CB* (Table 1). They correspond to 87,550,170–87,550,296, 87,536,457–87,536,541, 87,541,356–87,541,464, 87,549,347–87,549,518, 87,550,724–87,550,838, 87,566,069–87,566,241, 87,566,784–87,566,976, 87,595,765–87,595,814, 87,601,966–87,603,140, 87,605,645–87,605,775 on Chromosome 7 (Table 1). These positions could be responsible for the high frequency of AS in *ABCB1* pre-mRNA, leading to changes in *ABCB1* expression in these gene-deficient cell populations.

### Profile of the protein-protein interaction network linking the most differently expressed factors in KB-C2 cells deficient in *cdk6*

Transcriptome sequencing and quantification revealed that the knockout of *cdk6* gene (indicated by a red star in Fig. 4D) induced downregulation of KRAS, CDKN2B, NOTCH3, GADD45A, JUN, FOXO3, TGFBR1, and upregulation of CDKN2C, further induced downregulation of a series of factors, such as PDPK1, SERPINE1, PTGS2, MAPK10, RASSF5, PTGS2 and NGFR, and upregulation of PIK3R2, PIK3R3, and PRKACB (Fig. 4D). The majority of the signaling factors were downregulated. CD36, ITGA11, AGT, FGF21, INHBA and TGFA were the most significantly downregulated factors. FGF18, ACTN2, FGF11, GDF6, PIK3R3, PIK3R2 and MYH7 were the most significantly upregulated ones (Fig. 4D). Among the downregulated factors, KRAS, JUN, MAPK10, FN1 and SERPINE1 are in a relatively central position, and regulates multiple pathways (Fig. 4D). Among the upregulated factors, ATP2A2, PRKACB, PIK3R2, PIK3R3, and CXCL1 regulate multiple pathways (Fig. 4D).

**Fig. 5:**
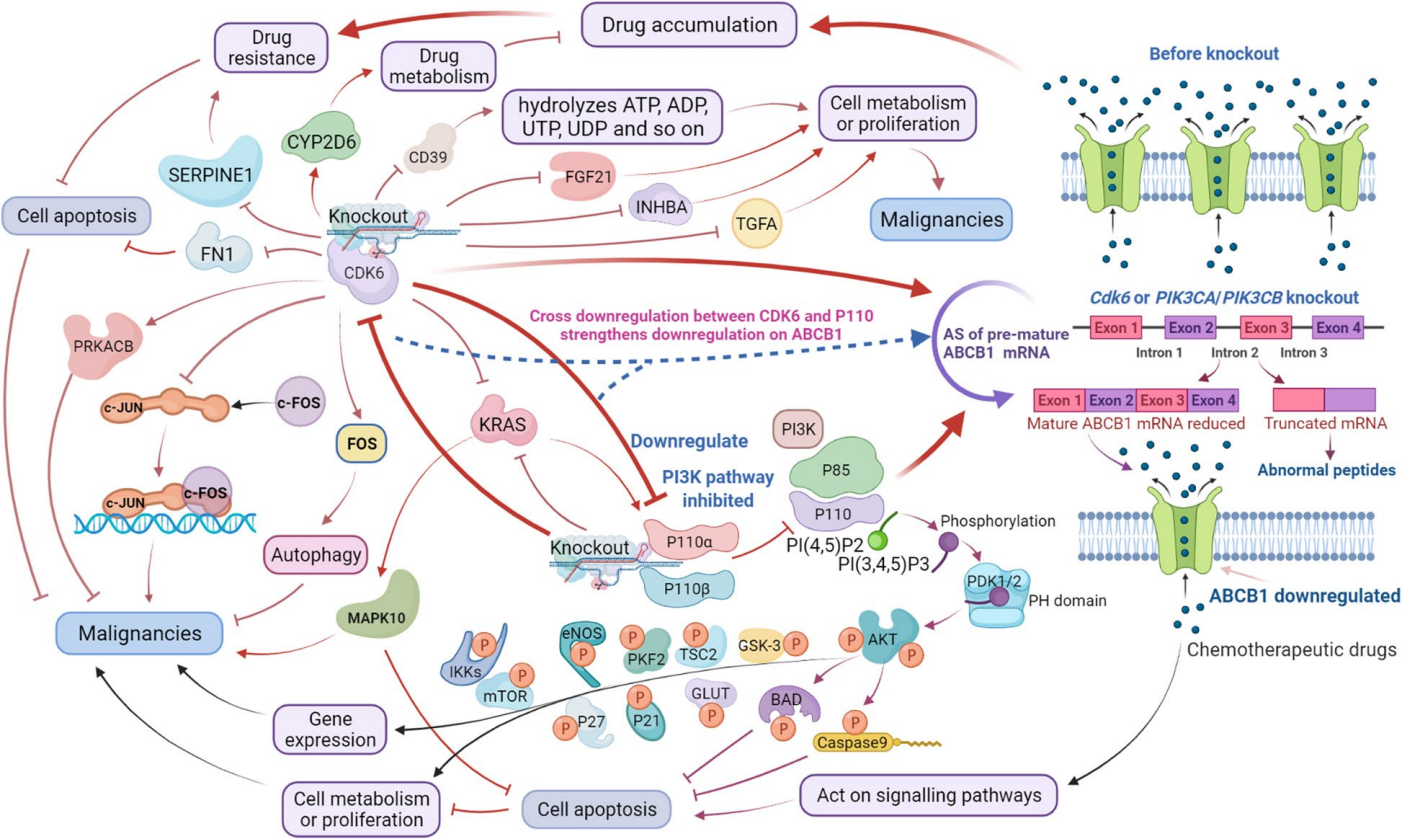
Regulation of CDK6-PI3K axis on ABCB1-mediated drug resistance. Knockout of *cdk6* gene in KB-C2 with CRISPR/Cas9 technique induced attenuation of ABCB1-mediated drug resistance. It promoted AS in the pre-mRNA expressing ABCB1, generating differential expression of the transcripts encoding functional ABCB1 protein or truncated abnormal peptides, further decreased the level of functional ABCB1 transporters. With the deficiency of *cdk6* in KB-C2 cells, PI3K 110α and 110β expression were downregulated, which induced AS of *ABCB1* pre-mRNA and led to inhibition of ABCB1 expression and reversal of ABCB1-mediated MDR, inhibiting cell division and promoting cell apoptosis. A series of signaling pathways critical for malignancies were downregulated in the KB-C2-k.o.cdk6 cell population

Among the significantly downregulated proteins, KRAS is a GTPase and a proto-oncogene product relaying early signals from outside the cell to the cell nucleus [39]. It is one of the effectors downstream of EGFR activation and triggers intracellular signaling cascades such as MAPK and PI3K [40]. It is interesting that KRAS was significantly downregulated in either CDK6 or P100α/P110β deficient cell populations. The JUN product, proto-oncoprotein c-JUN, in combination with c-Fos, forms oncogenic transcription factor that interacts with specific target DNA sequence to regulate gene expression and is involved in both translocations and deletions in human malignancies [41]. Mitogen-activated protein kinase 10 (MAPK10), also known as c-Jun N-terminal kinase 3 (JNK3) is a c-Jun N-terminal kinases (JNKs) that may prevent cell apoptosis. MAPK10/JNK3 signaling may induce carcinogenesis [42]. Knocking down MAPK10 suppressed ovarian cancer cell growth and migration [43]. Fibronectin 1 (FN1) is a glycoprotein of the extracellular matrix that binds to integrins (a type of membrane-spanning receptor proteins) [44]. It can suppress apoptosis and promoted viability, invasion, and migration in cancers [45]. *SERPINE1* gene in human encodes serpin E1, also known as endothelial plasminogen activator inhibitor or plasminogen activator inhibitor-1 (PAI-1), which has significant functions in the cancer occurrence, relapse and multidrug resistance [46]. Therefore, the knockout of *cdk6* gene inducing remarkable downregulation of these genes (Fig. 5) may contribute to the inhibition on the survivability or proliferation of the KB-C2 cells.

Among the significantly upregulated proteins, ATP2A2 as one of the P-type pumps important for higher order physiological processes, is an isoform of the SERCA Ca^2+^-ATPases that catalyze the hydrolysis of ATP, maintain nanomolar cytoplasmic Ca^2+^ levels, and provide high levels of Ca^2+^ in the endoplasmic reticulum, Golgi, and secretory vesicles which store Ca^2+^ for a wide range of signaling functions [47, 48]. cAMP-dependent protein kinase catalytic subunit beta (PRKACB) is a member of the serine/threonine protein kinase family and is a catalytic subunit of PKA. By activating the protein kinase A (PKA), which transduces the signal through phosphorylation of variant target proteins, it functions as a signaling molecule important for a variety of cellular activities [49]. As reported, down-regulation of PRKACB is related to the occurrence of various human malignancies [50]. the PI3K regulatory subunits PIK3R2/p85beta and PIK3R3/p55gamma, are frequently overexpressed in various cancers [51, 52]. The chemokine (C-X-C motif) ligand 1 (CXCL1) is a small peptide belonging to the CXC chemokine family. It promotes arteriogenesis through enhanced monocyte recruitment into the peri-collateral space and has relationship with tumor progression [53, 54]. For these significantly upregulated genes that have central regulation function and are involved in multiple signaling pathways (Fig. 4D), ATP2A2 and PRKACB upregulation may contribute to inhibiting the viability of KB-C2 cells. It is also the first time to unveil the significant upregulation of some malignancy related genes including PIK3R2, PIK3R3 and CXCL1. These results indicated that responsive upregulation or downregulation of a number of genes and signaling pathways occurred that affected multiple cell functions after *cdk6* knockout. The mostly changed signaling pathways downregulating ABCB1 expression and inhibiting cancer cell proliferation were concluded in Fig. 5.

### Knockout of *cdk6* or *cdk4* gene promotes apoptosis, but only *cdk6* deficiency reverses MDR in KB-C2 cells

**Fig. 6:**
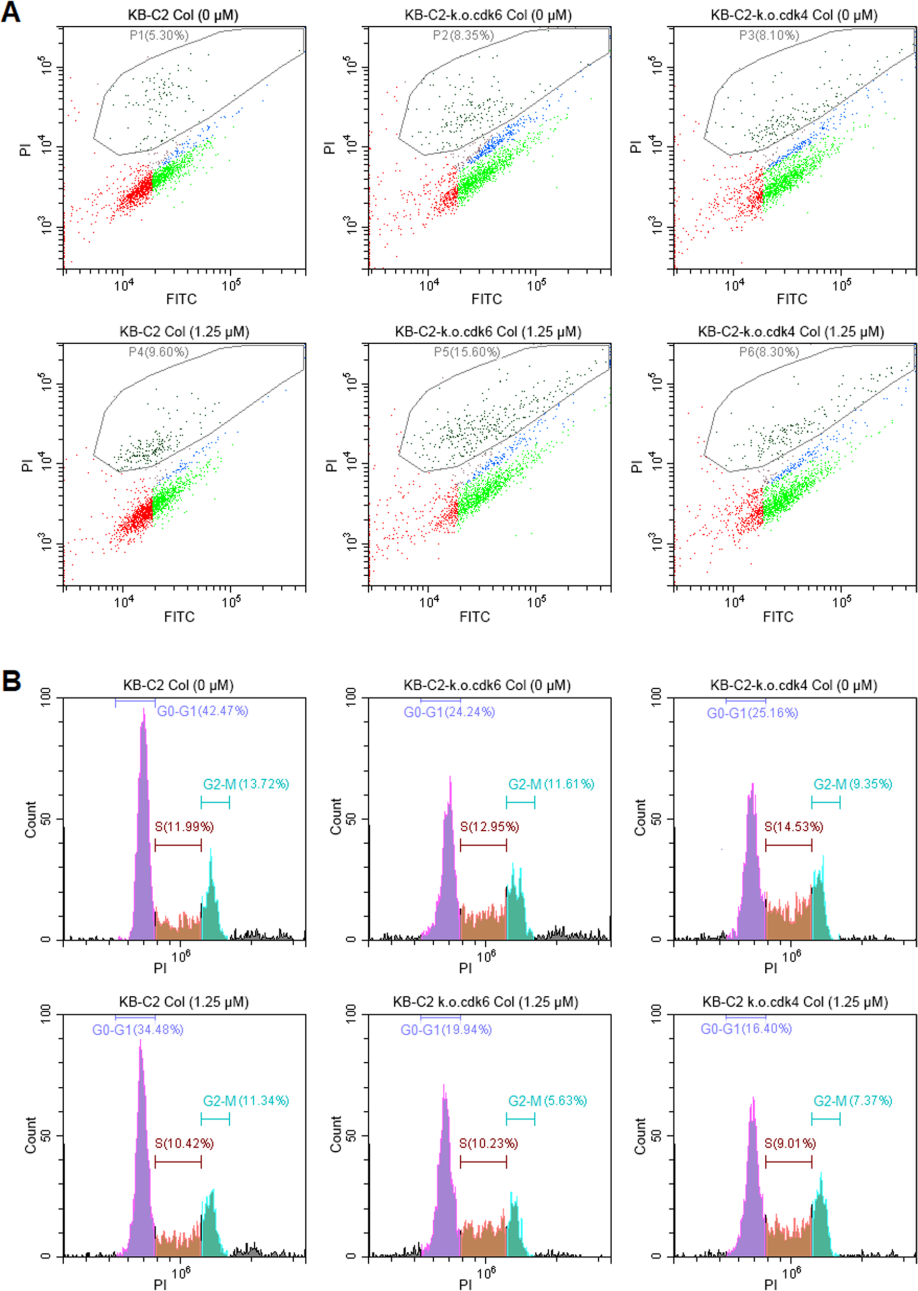
Flow cytometer analysis of cell apoptosis and cell cycle in KB-C2 cells with the deletion of *cdk6* or *cdk4* gene*.***A** Apoptosis of KB-C2 cells with deletion of *cdk6* or *cdk4* gene. The KB-C2-k.o.cdk6 and KB-C2-k.o.cdk4 cells were either incubated with a vehicle (media without colchicine) or colchicine (Col) for 24 h. The fluorescent intensity of the cells at the various heights on the axis are presented as spot groups showing the percentage of viable to severely apoptotic cell populations. The populations comprising heavily apoptotic (HA) cells (P1-P6), moderately apoptotic (MA) cells, slightly apoptotic (SA) cells and non-apoptotic (NA) cells were indicated by dots in black, blue, green, and red, sequentially. Col: colchicine. The fluorescence of PI and FITC was determined through PC7-A and FITC-A tracks, respectively. **B** Alteration of cell cycle in the KB-C2 cells with the deletion of *cdk6* or *cdk4* gene. The cells were co-incubated with the vehicle or colchicine (Col) for 24 h. The fluorescence of PI was determined through PE-A track

In the *cdk4-* or *cdk6*-deficient KB-C2 cells cultured with or without colchicine, the proportion of non-apoptotic cells was half declined compared with the original KB-C2 cells (Fig. 6A). The deletion of the *cdk6* gene induced transition of more cells from slightly apoptotic status to heavily apoptotic status (Fig. 6).

The culture of KB-C2 cells with colchicine (1.25 μM) only produced minor increase of apoptotic cells because KB-C2 cancer cells are highly resistant to colchicine (Fig. 6). However, *cdk6* knockout remarkably increased the drug sensitivity and induced significantly promoted transition from moderately apoptotic cells to heavily apoptotic cells (15.6%) in KB-C2-k.o.cdk6 cells incubated with colchicine compared with control cells incubated without colchicine (8.35%) (Fig. 6). Slightly increase of non-apoptotic cells and decrease of moderately apoptotic cells was detected in the drug-treated KB-C2-k.o.cdk4 cell populations (*cdk4* deleted KB-C2) compared with control cells (Fig. 6).

### The deletion of *cdk6* or *cdk4* accelerates cell senescence in the G_1_ phase, whereas *cdk6* deletion remarkably decreased the drug resistance of KB-C2 cells to colchicine

Deletion of either *cdk4* or *cdk6* accelerated senescence in G_1_ phase and promoted apoptosis of KB-C2 cells (Fig. 6B, Table S3﻿). However, the deficiency of *cdk6* but not *cdk4*, caused significant inhibition on cell division in G2-M phase in the presence of colchicine compared with non-gene-deficient KB-C2 cells. This further demonstrated that the knockout of *ckd6* gene but not *cdk4* reversed the MDR of KB-C2 via a mechanism of inhibiting cell division. As reported, colchicine binds to microtubules that form the mitotic spindle during cell division, thereby leads to mitotic arrest and ultimately cause cell death [55]. Downregulation of ABCB1 by the deficiency of CDK6 may reduce the efflux of colchicine which results in the increase of colchicine accumulation within the cells and enhanced inhibition on cell division.

### Loss of gene *cdk6* remarkably increased the sensitivity of KB-C2 tumors to DOX by increasing DOX accumulation inside the tumor cells

**Fig. 7:**
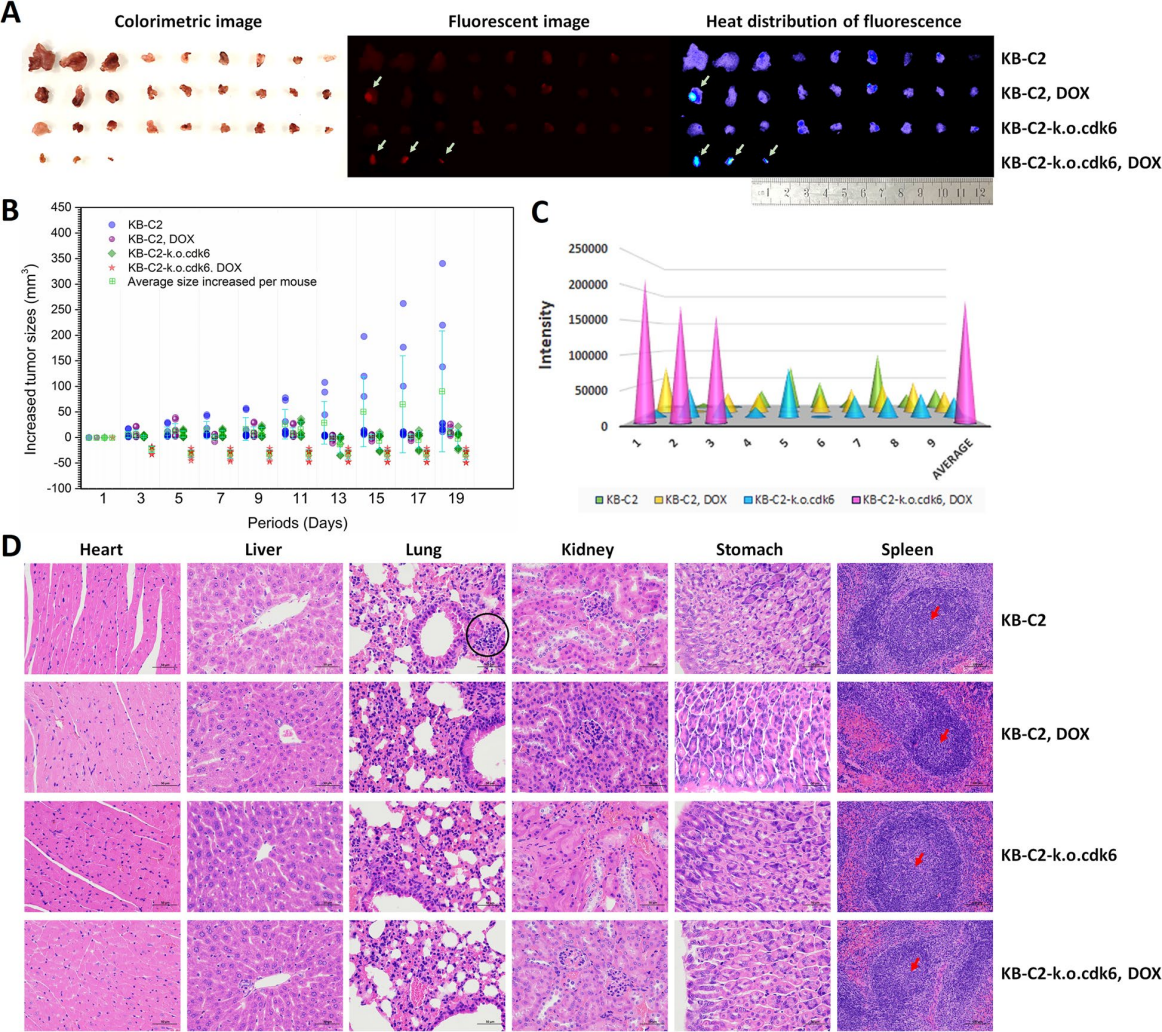
In vivo study of the function of gene *cdk6* to regulate drug-resistance of KB-C2 tumors. **A** Image of the tumors from the tumor-bearing mice showing the tumor morphology and the fluorescent DOX (depicted with yellow-green arrows) accumulated in the *cdk6*-present or *cdk6*-deficient tumors. The mice treated with saline were set as the blank control group. The tumors were sampled by the 19th day of DOX treatment. **B** Increased tumor sizes after the mice received chemotherapy using DOX. The mice treated with saline were set as the blank control group. **C** Comparison of the relative fluorescent intensity showing the difference of DOX accumulation in the *cdk6*-present or *cdk6*-deficient tumors. **D** Pathology of main organs of the mice bearing KB-C2 or KB-C2-k.o.cdk6 tumors. The organs were sampled by the 19th day after DOX treatment. The circle in black depicts focal infiltration of peri-bronchial inflammatory cells. The arrows in red depicts white pulp germinal center expansion, which occurred frequently in the tumor-bearing mice untreated with DOX, but only occasionally detected in the DOX-treated mice bearing KB-C2-k.o.cdk6

DOX remarkably inhibited the *cdk6* deficient KB-C2, i.e., KB-C2-k.o.cdk6 tumors. Among the 9 KB-C2-k.o.cdk6 tumors, 6 were completely eliminated, and only 3 with very small sizes were detected (Fig. 7A and B) by the 19th day after DOX treatment. Tumor enlargement was most rapid in DOX-untreated KB-C2 group, followed by DOX-treated KB-C2 and DOX-untreated KB-C2-k.o.cdk6 groups. This result indicated that loss of gene *cdk6* remarkably increased the sensitivity of KB-C2 tumors to DOX. Meanwhile, *cdk6* deficiency can inhibit tumor growth to some extent because this gene is important for cancer cell proliferation.

To find out the mechanisms of the enhanced inhibition in KB-C2-k.o.cdk6 tumors, the drug sensitivity to DOX was compared between the *cdk6*-present and *cdk6*-deficient tumors. All remaining KB-C2-k.o.cdk6 tumors showed strong fluorescence of DOX, while only a small part of the KB-C2 tumors had detectable DOX accumulation (Fig. 7C). This result demonstrated that *cdk6* deficiency greatly reversed the drug resistance caused by ABCB1, of which DOX is its substrate, as more amount of DOX was concentrated within the tumor cells where ABCB1 was downregulated by *cdk6* deficiency.

With the inhibition of tumor growth, pathological changes including focal infiltration of peri-bronchial inflammatory cells and expansion of the germinal center in spleen white pulp was remarkably reduced in the DOX-treated nude mice bearing KB-C2-k.o.cdk6, as compared with the KB-C2 groups or the DOX-untreated KB-C2-k.o.cdk6 group (Fig. 7D).

### Combining knockdown of *cdk6* and DOX-chemotherapy inhibited growth and metastasis of KB-C2 tumor in vivo

**Fig. 8:**
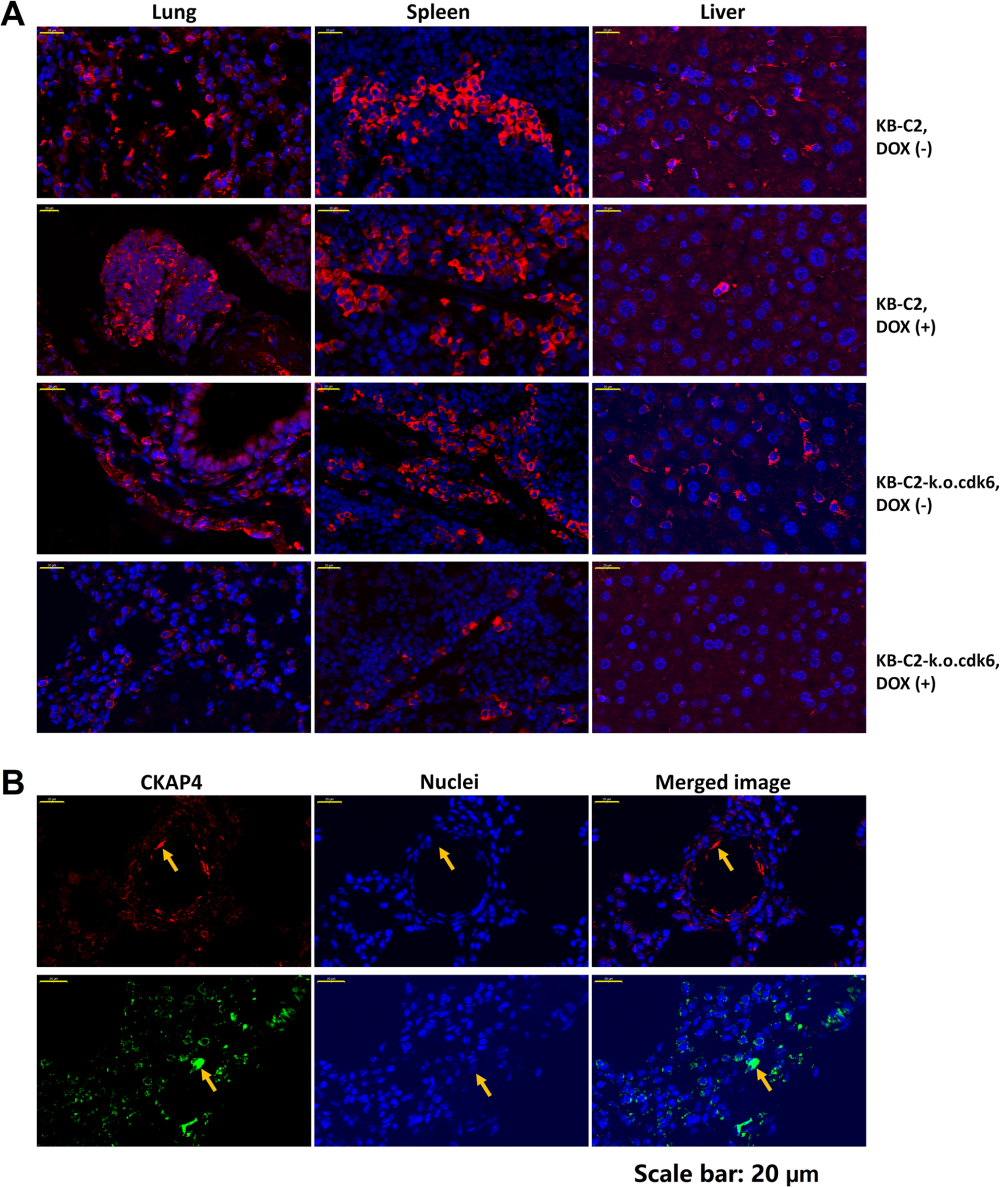
Migration of *cdk6* intact and deficient KB-C2 cells to lungs, spleens and livers in the nude mice. **A** The results of immunofluorescence labeling of CKAP4 showed that the *cdk6*-deleted cancer cells in the nude mice injected with DOX were the least in number of metastasis spots. DOX had little effect on the metastasis of cancer cells without *cdk6* deletion, and had a significant effect on the metastasis of *cdk6*-deleted cancer cells. All nude mice without DOX injection had serious cancer cell metastasis. **B** KB-C2-k.o.cdk6 cells were eliminated from the lungs in nude mice after treatment with DOX. The arrows in orange depict the distorted dead KB-C2-k.o.cdk6 cells that had expressed high level of CKAP4. Due to the damage of the nuclei, DAPI fluorescence was absent in these cells. To testify this phenomenon, red and green fluorescence, respective of Cy3 and FITC labeled secondary antibodies, were used to determine antigen CKAP4-abundant KB-C2-k.o.cdk6 cells

Gene differential expression analysis and immunofluorescence analysis indicated that KB-C2 cells with small cell nuclei and spindle-shaped morphology showed strong CKAP4-positive signal. High levels of tumor cell metastasis were detected in the liver, lung and spleen of KB-C2 tumor-bearing nude mice, and more tumor cells were distributed inside the organs, as compared with KB-C2-k.o.cdk6 tumor- bearing nude mice (Fig. 8). In KB-C2-bearing nude mice treated with DOX, the tumor cells in the organs were only slightly less than those without DOX treatment, and in the lungs, the tumor cells showed obvious inward invasion and growth. In KB-C2-k.o.cdk6-bearing nude mice with DOX treatment, the tumor cells that had invaded in the interior of the organ were the least (Fig. 8), and for the lungs, they were detected only around one outside and no apparent signals were detected inside the lung tissues. These results demonstrated that combining knockdown of *cdk6* and DOX chemotherapy inhibited the metastatic ability of MDR KB-C2 cells. Interestingly, KB-C2-k.o.cdk6 cells were eliminated in the lungs of the nude mice after they received DOX by injection.

## Discussion

MDR of cancers can induce failure of chemotherapy. As one major reason of MDR in cancers, ABC transporters are highly expressed in various MDR cancer cell lines [56, 57]. Today, a number of ABC transporter inhibitors have been explored to attenuate the activity of the ABC transporters to pump out chemotherapeutic drugs [2, 58]. However, most of the inhibitors may have several targets, some of which have important function in normal cells [31]. Furthermore, the regulatory mechanisms are greatly unknown; as such risks of side-effects may be increased. Seeking safer targets, often based on tumor-specific proteins that regulate ABC transporters, has become an ideal approach to improve MDR-reversal in cancer therapy. As ABCB1 is a vital ABC transporter for the cancer cells to extrude a variety of anti-cancer drugs [31, 59, 60], we investigated the targets to reverse ABCB1-mediated MDR based on screening of effective reversal agents and gene deletion technology, and revealed a novel targeting pathway. In KB-C2, CDK6 may induce on-off regulation of ABCB1 expression through coordination with PI3K 110α/β and inducing AS in the *ABCB1* pre-mRNA. In a parallel study, similar regulation of ABCB1-mediated MDR by CDK6 was observed in H460/MX80-k.o.*cdk6*, in which *cdk6* was knocked out from the NSCLC H460/MX80 MDR cells (Fig. S3). Therefore, CDK6 may be potential and secure targets for reversing ABCB1-mediated MDR, since its overexpression has been shown to stimulate cancer cell proliferation [61].

At present, the project focusing on the function of ribociclib in inhibiting the ABCB1 mediated-MDR in cancers are undergoing in our laboratory. Because the mechanisms for ribociclib to downregulate ABCB1 and inhibit P-gp mediated MDR in cancers could be associated with many aspects, we are currently performing experiments, including real-time PCR of ABCB1 in ribociclib treated cells, or transcriptome sequencing and quantification, in addition to Western blot and immunofluorescence which we have done, to explore the ABCB1 expression at the transcription level. We will also try to explore other potential pathway(s) through the participation of which, ribociclib might downregulate ABCB1 expression. In addition, our unpublished data showed that the structure of ABCB1 could be changed by ribociclib at effective MDR-reversal concentration (e.g., 9 μM) under physiology condition, causing inhibition of the ATPase and drug-efflux activity of ABCB1, indicating that ABCB1 was less stable through interaction with ribociclib. Due to the intricacy of the function of ribociclib within the MDR cancer cells, specific gene deletion was performed to analyze whether CDK6 could be an effective target for overcoming ABCB1-mediated MDR in cancer cells.

We recently realized that the knockout of mutant PI3K 110α or 110β achieved inhibition of ABCB1 expression as well as reversal of MDR mediated by ABCB1 [2]. We further found in this study that downregulation of CDK6 expression through gene knockout downregulated the expression of PI3K P110α and P110β subunits, and vice versa. This cross regulation between CDK6 and P110α/P110β could explain the remarkable decrease in ABCB1 expression and MDR in *cdk6* or P110α/P110β deleted KB-C2 cells.

Upon further analysis of the regulatory mechanism of ABCB1 expression by CDK6, CDK4 and PI3K 110 α/β, we surprisingly found that the TPM levels of the ABCB1 transcripts expressing the ABCB1 protein and truncated ABCB1 peptides varied significantly between the gene-deficient KB-C2 cell populations and non-gene-deficient KB-C2 cells. In addition, we found that a high frequency of SE splicing events had occurred during AS of the *ABCB1* pre-mRNA that expresses ABCB1. This could explain the markedly reduced ABCB1 level in KB-C2-k.o.cdk6 cells and enhanced expression of ABCB1 or its isoform in KB-C2-k.o.cdk4 cells, which resulted in different efficacy on ABCB1-mediated MDR. Interestingly, a knockout experiment indicated that *cdk6* was downregulated along with the knockout of *cdk4* in KB-C2 cells. To our knowledge, it is the first time that the responsive AS occurrence in the pre-mRNA of *ABCB1* was found to be induced by the deletion of *cdk6*, *cdk4* or PI3K 110α/β subunits. This will surely have significance in reversing ABCB1-mediated MDR by modulating *cdk6* or *cdk4* gene expression.

CDK6 has been suggested to be a redundant homolog of CDK4 in the past [62]. For the first time, our study demonstrated that CDK6 and CDK4 may be two homologous proteins that differentially regulate ABCB1 expression in MDR KB-C2 cells. Indeed, our results indicated that the knockout of *cdk6* reversed ABCB1-mediated MDR in KB-C2 cells (Fig. 2, Fig. 6). In this study, differential gene expression revealed that, along with the deletion of CDK6, a series of signaling pathways altered, for example, JUN/MAPK10 which is crucial in regulating tumorigenesis [42, 63], NOTCH3 which is involved in carcinoma and immune tolerance [64], were downregulated in KB-C2-k.o.cdk6 cell population, whereas CYP2D6 which was responsible for the metabolism of many drugs [65], was upregulated. Combining our findings that *cdk6*-deficient cancer cells lost ABCB1-mediated MDR via co-downregulating PI3K 110α/β, we deem that *cdk6* could be a promising target for reversing ABCB1-mediated MDR and inhibiting cancer cell proliferation simultaneously. It may be hopeful to develop strategies based on targeting CDK6 for combined chemotherapy of MDR cancers with improved accuracy. In this aspect, modernized functional biomaterials can be integrated for further enhancing the targeting inhibition on CDK6 in cancer cells.

In summary, we demonstrated that regulation of ABCB1 can be realized through targeting CDK6-PI3K axis. Knockout of the *cdk6* gene unveiled a novel targeting pathway for overcoming ABCB1-mediated MDR in cancers. PI3K 110α/β signaling was downregulated in the *cdk6*-deficient KB-C2 cell populations. A high frequency of responsive AS, mainly SE events, were induced by CDK6, CDK4, and PI3K 110α/β level changes, and these AS events finally led to the alteration of the ABCB1 expression and ABCB1-mediated MDR in *cdk6* or *cdk4* deficient KB-C2 cancer cells. Cross-regulation between CDK6 and PI3K110α/110β was discovered, and knockout of either one of these two genes could induce downregulation of the other and remarkable inhibition of ABCB1 expression, implying that they may synergize with each other to regulate ABCB1 expression. This will greatly strengthen the efficiency during reversing ABCB1 when using either of them as target.

With the knockout of *cdk6*, quite a few of signaling factors leading to inhibition of cancer cell proliferation were found to be promoted. For example, FOS-promoting cell autophagy [38] was upregulated. It is possible that these genes are quite variable in expression to adapt to CDK6 deficiency.

In the Western blot analysis, the positive signal band showed certain peptide with a reduced molecular weight in the *cdk4* or *cdk6* deficient cell populations. This should be induced by DNA repairment during CRISPR/Cas9 gene editing, that is, truncated proteins with similarity to wild-type CDK4 or CDK6 was produced after chromosomal repairment. To confirm this, we performed RT-PCR and certified deficiency of the objective region in *cdk4* or *cdk6* genes on chromosomes, and the results demonstrated knockout of wild-type *cdk4* or *cdk6* genes.

In vivo experiment demonstrated the function of *cdk6* in maintaining ABCB1-mediated drug resistance in cancers. Since *cdk6* deficiency or PIK3CA/PIK3CB deletion downregulated each other, and PIK3CA/PIK3CB knockout also downregulated ABCB1, we deem that CDK6-PI3K signaling axis is an efficient target for attenuating ABCB1-mediated MDR. To some degree, *cdk6* deficiency inhibited KB-C2 tumor growth, which showed similar efficacy with DOX treated KB-C2 tumors. However, the efficacy was far less than the DOX-treated KB-C2-k.o.cdk6 tumor group. ABCB1-mediated drug resistance appeared to be a dominant barrier for inhibition on KB-C2 tumor.

Most known cancer cells have increased PIK3CA expression or more active PIK3CA which may lead to enhanced cell survivability, proliferative or anti-apoptosis ability, and overexpression of ABCB1 as we reported [2]. therefore, realizing downregulation of PIK3CA and ABCB1 by knocking out CDK6 in MDR cancer cells overexpressing ABCB1 might benefit combined chemotherapy against cancer cells with ABCB1-mediated MDR.

Through transcriptome analysis in this study, we found that protein CKAP4 is expressed at especially high level in both KB-C2 and KB-C2-k.o.cdk6 cells. It was reported that CKAP4 was minimally expressed in non-tumor cells [66], therefore, it was adopted as a biomarker for investigation of KB-C2 and KB-C2-k.o.cdk6 metastasis in the tissues of nude mice. We performed transcriptome sequencing and quantification to test the protein expression at the transcription level. The results reported that CDK6 expression in MDR KB-C2 cells (over expressing ABCB1, Fig. S4) was elevated to approximately 7 folds (Fig. S5) compared to drug sensitive parental KB-3-1 cells which did not express detectable amount of ABCB1 (Fig. S4). This provided a clue for clarifying the regulatory effect of CDK6 on ABCB1 by specific gene knockout experiment. By knocking down *cdk6* in KB-C2 cell population, metastasis and survival of KB-C2-k.o.cdk6 tumor cells were remarkably inhibited in the DOX-treated nude mice, mainly because the drug resistance of KB-C2-k.o.cdk6 was inhibited compared with KB-C2.

Although a combination of prospective and retrospective cohort study involving 131 cases of advanced stage invasive breast cancer (which have received neoadjuvant chemotherapy) indicated that the expression of ABCB1 had no significant statistical correlation to metastases (*p* = 0.659) [67], whether ABCB1 has any effect on tumor cell migration, which has not been clearly studied, is an undergoing project we are studying.

Based on these findings, combined targeted chemotherapy with improved efficacy can be realized through downregulating CDK6-PI3K signaling axis in cancers overexpressing ABCB1, which is a common ABC member overexpressed in most malignant tumors and functions to generate drug resistance by efflux of its substance drugs.

## Conclusion

In conclusion, we deem that CDK6-PI3K axis can be ideal target for reversing ABCB1 mediated MDR in cancers without inducing cancer cell proliferation, and this finding is of great significance for the combined anticancer chemotherapy which can reverse multidrug resistance and inhibit the growth of tumor cells at the same time. This study revealed the cross downregulation between CDK6 and PI3K in *cdk6* deficient cancer cell populations, demonstrated that CDK6-PI3K axis could be new target for inhibiting ABCB1-mediated MDR. Meanwhile, knockout of *cdk6* could inhibit cancer cell proliferation and malignancy. These new findings will surely benefit exploration of new drugs targeting *cdk6* or *PIK3CA/PIK3CB* genes or gene products through which the therapeutic effect could be optimized.

## Supplementary Information

**Additional file 1: Fig. S1.** Network and the interactions of the Top10 differentially expressed genes involved in KEGG Endocrine resistance. **A** Network of enriched terms colored by cluster ID, where nodes that share the same cluster ID are typically close to each other. **B** Protein-protein interaction network and components identified in the gene lists. String platform was used for the analysis.

**Additional file 2: Fig. S2.** Protein-protein interaction of the proteins which are most correlated with endocrine resistance.

**Additional file 3: Fig. S3.** Regulation of CDK6 on ABCB1-mediated MDR in H460/MX80. **A** RT-PCR certification of *cdk6* or *cdk4* gene knockout in H460/MX80 cells. The cells were transfected with the CRISPR/Cas9 all-in-one plasmid and stabilized for 2 weeks and then analyzed for the *cdk4* or *cdk6* mRNA amounts. **B** MTT analysis of cell viability of MDR H460/MX80 cells (with very low level of ABCB1 expression) and the *cdk6*-deficient H460/MX80 cells, i.e., H460/MX80-k.o.cdk6 cells. Paclitaxel and colchicine, which are ABCB1 substates, were used for co-culture with H460/MX80 and H460/MX80-k.o.cdk6 cells for 72 h.

**Additional file 4: Fig. S4.** Upregulation of ABCB1 in MDR KB-C2 cells compared to KB-3-1 cells. Transcriptome sequencing and quantification were performed in the MDR KB-C2 cells and drug sensitive parental KB-3-1 cells in the same condition, and the data showing the level of all the *ABCB1* transcripts is summarized in the graph. As indicated by the transcripts per million mapped reads (TPM) value, the number of full-length-*ABCB1* transcripts (mRNA) in KB-C2 cells were substantially increased as compared with that in KB-3-1 cells. The transcripts ENST00000265724, ENST00000622132 and MSTRG.27385.1 corresponding to the full-length of ABCB1 protein were indicated by *.

**Additional file 5: Fig. S5.** Upregulation of CDK6 in MDR KB-C2 cells compared to KB-3-1 cells. Transcriptome sequencing and quantification were performed in the MDR KB-C2 cells and drug sensitive parental KB-3-1 cells in the same condition, and the data showing the level of all the *cdk6* transcripts is summarized in this graph. As indicated by the transcripts per million mapped reads (TPM) value, the number of full-length-*cdk6* transcripts (mRNA) in KB-C2 cells were 7-folds of that in KB-3-1 cells. The transcripts ENST00000265734 and ENST00000424848 corresponding to the full-length of CDK6 protein were indicated by *.

**Additional file 6: Table S1.** Differential expression of CDK6, ABCB1, CDK4, PIK3CA, PIK3CB and GAPDH genes in KB-C2 and KB-C2-k.o.cdk6. Identical amounts of total RNA from KB-C2 and KB-C2-k.o.cdk6 cells were analyzed for transcriptome sequences and differential gene expression. The transcripts encoding full or effective length of proteins are listed. 

**Additional file 7: Table S2.** Downregulation of the expression level of CDK6 in KB-C2-k.o.110α, KB-C2-k.o.110β, MX80-k.o.110α, or MX80-k.o.110β populations.

**Additional file 8: Table S3.** The percentages of non-apoptotic cells in the different phases of the cell cycle. The data were calculated based on the results in the histograms indicating a representative cell cycle (Fig. 6).

## Data Availability

Sequence information of *cdk4* (NM_000075.3) and *cdk6* (NM_001259.7) are available in GenBank. All other data are available in the main text or the supplementary materials. The datasets used and/or analyzed during the current study are available from the corresponding author on reasonable request.

## Abbreviations

ABC transporter: ATP-binding cassette (ABC) transporter
ACTN2: Alpha-actinin 2
AF488: Alexa Fluor 488
AGE: Advanced glycation end product
AGT: Angiotensinogen
AKT/PKB: Protein kinase B
ATP2A2: ATPase sarcoplasmic/endoplasmic reticulum Ca^2+^ transporting 2
ATP5ME: Mitochondrial ATP synthase subunit e
ATP5PF: Mitochondrial ATP synthase-coupling factor 6
ATP6V1B2: Brain V-type proton ATPase subunit B2
AS: Alternative splicing
BCRP/ABCG2: Breast cancer resistance protein
CCNA2: Cyclin A2 gene
MET: Mesenchymal to epithelial transition factor, a hepatocyte growth factor receptor tyrosine kinase
CDKN2B: Cyclin dependent kinase inhibitor 2B
CDKN2C: Cyclin dependent kinase inhibitor 2C
CDK4: Cyclin dependent kinase 4
*cdk4*: Gene of CDK4
CDK6: Cyclin dependent kinase 6
*cdk6*: Gene of CDK6
CDKs: Cyclin dependent kinases
CD36: Cluster of differentiation 36
CKAP4: Cytoskeleton-associated protein 4
CRISPR: Clustered regularly interspaced short palindromic repeats
CXCL1: Chemokine (C-X-C motif) ligand 1
CYP2D6: Cytochrome P450 family 2 subfamily D member 6
DAPI: 4′,6-diamidino-2-phenylindole
DEG: Differential expression genes
DMSO: Dimethyl sulfoxide
EC_50_: Concentration for 50% of maximal effect
EGFR: Epidermal growth factor receptor
EMT: Epithelial-mesenchymal transition
ERK: Extracellular regulated protein kinase
E2F: E2 transcription factor
FAT1: FAT atypical cadherin 1
FBS: Fatal bovine serum
FDA: Food and Drug Administration (FDA)
FGF11: Fibroblast growth factor 11
FGF18: Fibroblast growth factor 18
FGF21: Fibroblast growth factor 21
FLT3-ITD: Fms-like tyrosine kinase 3 - in-frame internal tandem duplications
FN1: Fibronectin 1
FOS: Fos proto-oncogene, AP-1 transcription factor subunit
FOXO3: Forkhead box O3
FRKM: Fragments per kilobase of exon per million mapped reads
GADD45A: Growth arrest and DNA damage inducible alpha
GAPDH: Glyceraldehyde-3-phosphate dehydrogenase
GDF6: Growth differentiation factor 6
GLI2: GLI family zinc finger 2
GPER1: G-protein coupled estrogen receptor 1
HCK: SRC family kinase HCK
*HRP*: Horseradish peroxidase
IC_50_: 50% inhibiting concentration
IF: Immune-fluorescence
INHBA: Inhibin subunit beta A
ITGA11: Integrin subunit alpha 11
JNK: c-Jun N-terminal kinase
JUN: Jun proto-oncogene, AP-1 transcription factor subunit
KEGG: Kyoto Encyclopedia of Genes and Genomes
MAP K10: Mitogen-activated protein kinase 10
MATS: Multivariate analysis of transcript splicing
MDR: Multidrug resistance
MEK: Mitogen-activated extracellular signal-regulated kinase
miRNA: micro RNA
MYH7: Myosin heavy chain 7
NBDs: Nucleotide-binding domains
Neu2: Cytosolic sialidase
NF-κB: Facilitates nuclear factor kappaB
NGFR: Nerve growth factor receptor
NOTCH3: Neurogenic locus notch homolog protein 3
NOTCH2NLB: Notch 2 N-terminal like B
ORF: Open reading frame
PDPK1: 3-phosphoinositide dependent protein kinase 1
P-gp: P-glycoprotein
PI3K: Phosphoinositide 3-kinase
P110α: PI3K subunits PIK3CA
P110β: PI3K subunits PIK3CB
Raf: Rapidly accelerated Fibrosarcoma
PIK3R2: Phosphatidylinositol 3-kinase regulatory subunit beta
PIK3R3: Phosphatidylinositol 3-kinase regulatory subunit gamma
PKA: Protein kinase A
PPI: Protein-protein interaction
PRKACB: cAMP-dependent protein kinase catalytic subunit beta
PTGS2: Prostaglandin-endoperoxide synthase 2
RAGE: AGE receptor
Ras: Rat sarcoma
RASSF5: Ras association domain family member 5
Rb: Retinoblastoma protein
ROS: Reactive oxygen species
SE: Skipped exon
SgRNA: Single guide RNA
SERCA: Sarco/endoplasmic reticulum Ca^2+^-ATPases
SERPINE1: Serpin family E member 1
SOX2: SPY homology box 2
TGFA: Transforming growth factor alpha
TGFBR1: Transforming growth factor beta receptor 1
TMDs: Transmembrane domains
TPM: Transcripts per million mapped reads
