# Supplementary material for: CDK6-PI3K signaling axis is an efficient target for attenuating ABCB1/P-gp mediated multi-drug resistance (MDR) in cancer cells
Source: Mol Cancer. 2022 Apr 22;21:103. doi: 10.1186/s12943-022-01524-w (PMC9027122; doi:10.1186/s12943-022-01524-w)
Supplement: Supplementary file 1 — Additional file 1: Fig. S1. Network and the interactions of the Top10 differentially expressed genes involved in KEGG Endocrine resistance. A Network of enriched terms colored by cluster ID, where nodes that share the same cluster ID are typically close to each other. B Protein-protein interaction network and components identified in the gene lists. String platform was used for the analysis. [file 12943_2022_1524_MOESM1_ESM.docx]

**
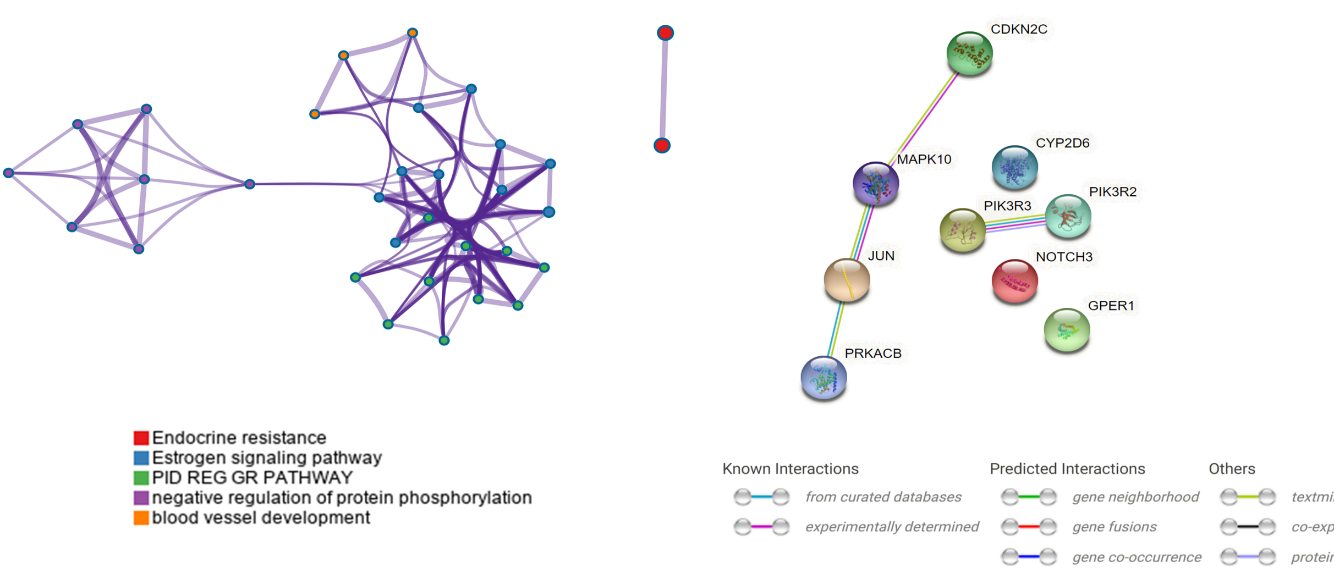
**

**Fig. S1 Network and the interactions of the Top10 differentially expressed genes involved in KEGG Endocrine resistance.** **A** Network of enriched terms colored by cluster ID, where nodes that share the same cluster ID are typically close to each other. **B** Protein-protein interaction network and components identified in the gene lists. String platform was used for the analysis.
