# Supplementary material for: CDK6-PI3K signaling axis is an efficient target for attenuating ABCB1/P-gp mediated multi-drug resistance (MDR) in cancer cells
Source: Mol Cancer. 2022 Apr 22;21:103. doi: 10.1186/s12943-022-01524-w (PMC9027122; doi:10.1186/s12943-022-01524-w)
Supplement: Supplementary file 2 — Additional file 2: Fig. S2. Protein-protein interaction of the proteins which are most correlated with endocrine resistance. [file 12943_2022_1524_MOESM2_ESM.docx]

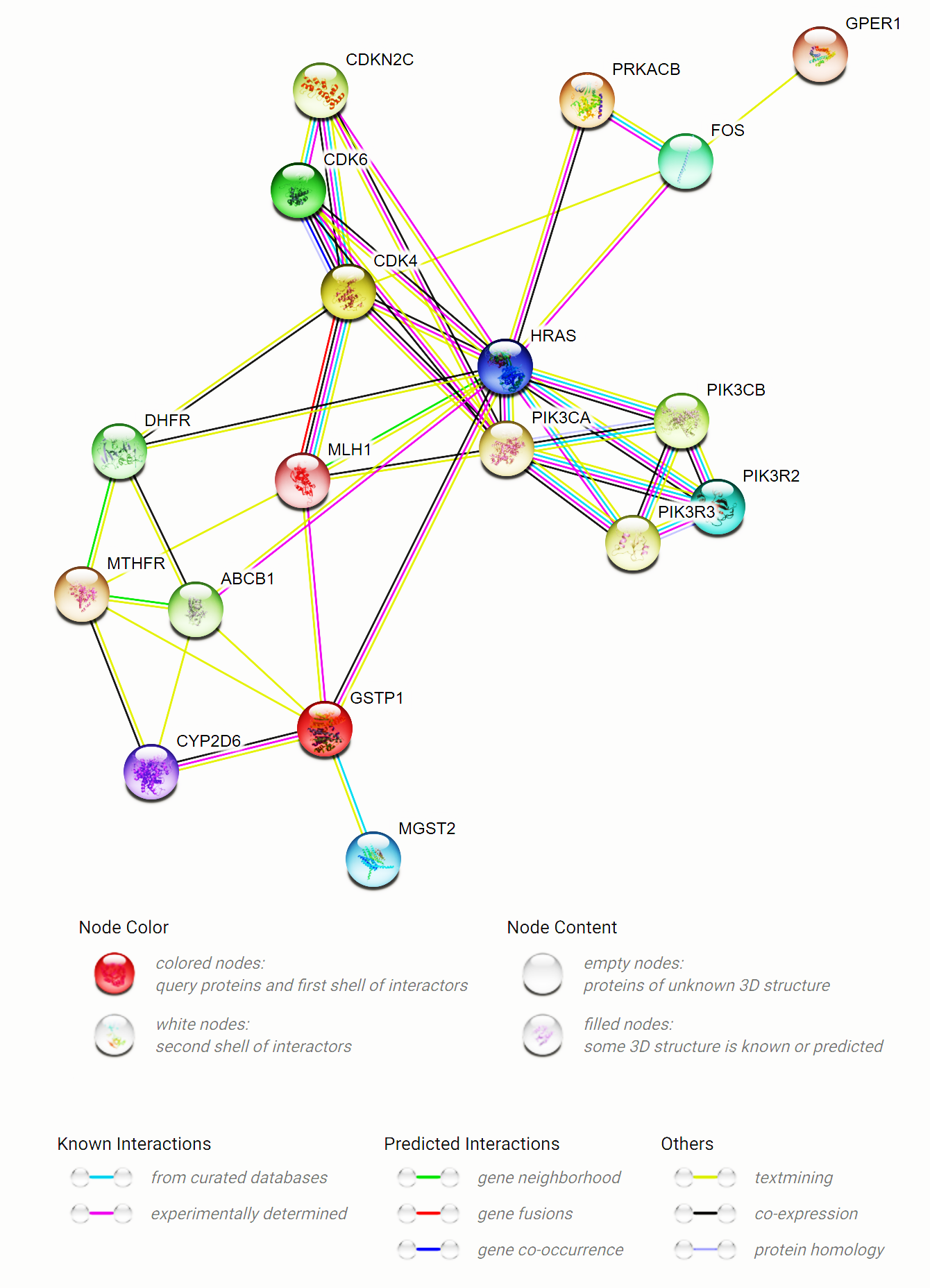


**Fig. S2 Protein-protein interaction of the proteins which are most correlated with endocrine resistance.**
