# Supplementary material for: CDK6-PI3K signaling axis is an efficient target for attenuating ABCB1/P-gp mediated multi-drug resistance (MDR) in cancer cells
Source: Mol Cancer. 2022 Apr 22;21:103. doi: 10.1186/s12943-022-01524-w (PMC9027122; doi:10.1186/s12943-022-01524-w)
Supplement: Supplementary file 3 — Additional file 3: Fig. S3. Regulation of CDK6 on ABCB1-mediated MDR in H460/MX80. A RT-PCR certification of cdk6 or cdk4 gene knockout in H460/MX80 cells. The cells were transfected with the CRISPR/Cas9 all-in-one plasmid and stabilized for 2 weeks and then analyzed for the cdk4 or cdk6 mRNA amounts. B MTT analysis of cell viability of MDR H460/MX80 cells (with very low level of ABCB1 expression) and the cdk6-deficient H460/MX80 cells, i.e., H460/MX80-k.o.cdk6 cells. Paclitaxel and colchicine, which are ABCB1 substates, were used for co-culture with H460/MX80 and H460/MX80-k.o.cdk6 cells for 72 h. [file 12943_2022_1524_MOESM3_ESM.docx]

**
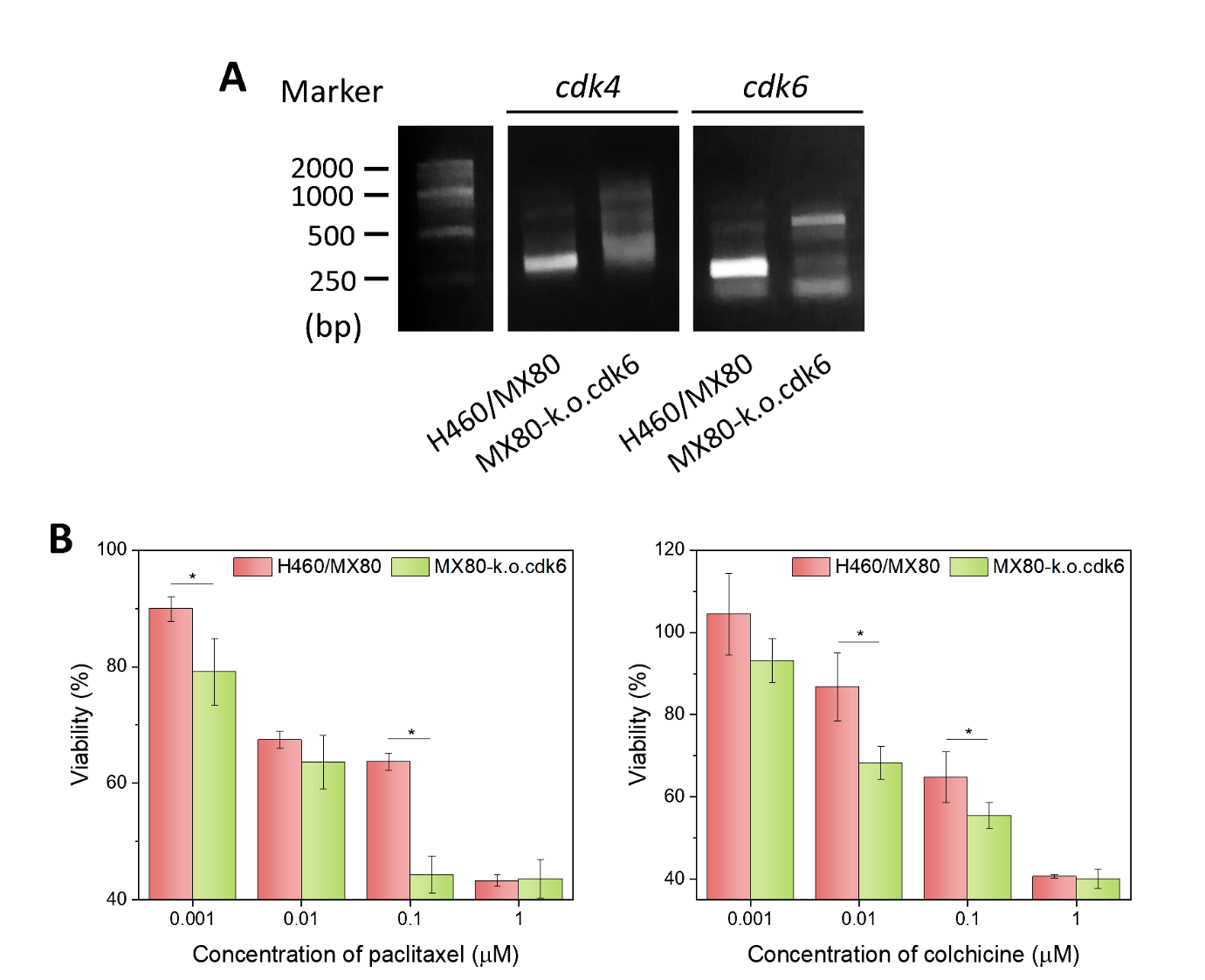
**

**Fig. S3 Regulation of CDK6 on ABCB1-mediated MDR in H460/MX80. A** RT-PCR certification of *cdk6* or *cdk4* gene knockout in H460/MX80 cells. The cells were transfected with the CRISPR/Cas9 all-in-one plasmid and stabilized for two weeks and then analyzed for the *cdk4* or *cdk6* mRNA amounts. **B** MTT analysis of cell viability of MDR H460/MX80 cells (with very low level of ABCB1 expression) and the *cdk6*-deficient H460/MX80 cells, i.e., H460/MX80-k.o.cdk6 cells. Paclitaxel and colchicine, which are ABCB1 substates, were used for co-culture with H460/MX80 and H460/MX80-k.o.cdk6 cells for 72 h.
