# Supplementary material for: CDK6-PI3K signaling axis is an efficient target for attenuating ABCB1/P-gp mediated multi-drug resistance (MDR) in cancer cells
Source: Mol Cancer. 2022 Apr 22;21:103. doi: 10.1186/s12943-022-01524-w (PMC9027122; doi:10.1186/s12943-022-01524-w)
Supplement: Supplementary file 4 — Additional file 4: Fig. S4. Upregulation of ABCB1 in MDR KB-C2 cells compared to KB-3-1 cells. Transcriptome sequencing and quantification were performed in the MDR KB-C2 cells and drug sensitive parental KB-3-1 cells in the same condition, and the data showing the level of all the ABCB1 transcripts is summarized in the graph. As indicated by the transcripts per million mapped reads (TPM) value, the number of full-length-ABCB1 transcripts (mRNA) in KB-C2 cells were substantially increased as compared with that in KB-3-1 cells. The transcripts ENST00000265724, ENST00000622132 and MSTRG.27385.1 corresponding to the full-length of ABCB1 protein were indicated by *. [file 12943_2022_1524_MOESM4_ESM.docx]

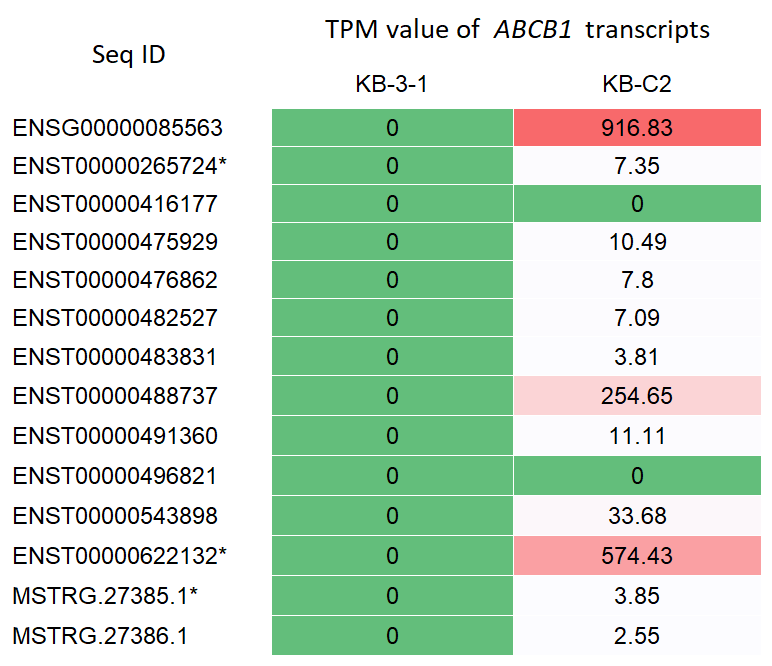


**Fig. S4 Upregulation of ABCB1 in MDR KB-C2 cells compared to KB-3-1 cells.** Transcriptome sequencing and quantification were performed in the MDR KB-C2 cells and drug sensitive parental KB-3-1 cells in the same condition, and the data showing the level of all the *ABCB1* transcripts is summarized in the graph. As indicated by the transcripts per million mapped reads (TPM) value, the number of full-length-*ABCB1* transcripts (mRNA) in KB-C2 cells were substantially increased as compared with that in KB-3-1 cells. The transcripts ENST00000265724, ENST00000622132 and MSTRG.27385.1 corresponding to the full-length of ABCB1 protein were indicated by *.
