# Supplementary material for: CDK6-PI3K signaling axis is an efficient target for attenuating ABCB1/P-gp mediated multi-drug resistance (MDR) in cancer cells
Source: Mol Cancer. 2022 Apr 22;21:103. doi: 10.1186/s12943-022-01524-w (PMC9027122; doi:10.1186/s12943-022-01524-w)
Supplement: Supplementary file 5 — Additional file 5: Fig. S5. Upregulation of CDK6 in MDR KB-C2 cells compared to KB-3-1 cells. Transcriptome sequencing and quantification were performed in the MDR KB-C2 cells and drug sensitive parental KB-3-1 cells in the same condition, and the data showing the level of all the cdk6 transcripts is summarized in this graph. As indicated by the transcripts per million mapped reads (TPM) value, the number of full-length-cdk6 transcripts (mRNA) in KB-C2 cells were 7-folds of that in KB-3-1 cells. The transcripts ENST00000265734 and ENST00000424848 corresponding to the full-length of CDK6 protein were indicated by *. [file 12943_2022_1524_MOESM5_ESM.docx]

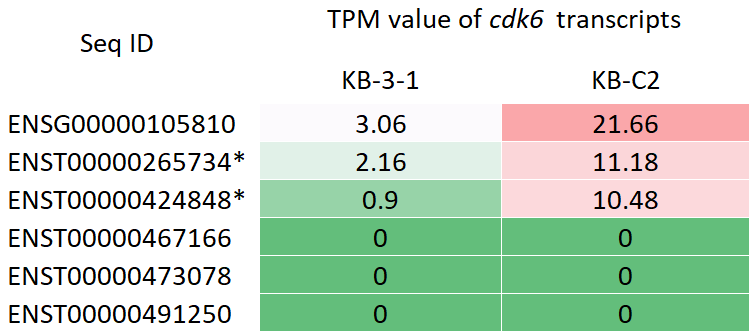


**Fig. S5 Upregulation of CDK6 in MDR KB-C2 cells compared to KB-3-1 cells.** Transcriptome sequencing and quantification were performed in the MDR KB-C2 cells and drug sensitive parental KB-3-1 cells in the same condition, and the data showing the level of all the *cdk6* transcripts is summarized in this graph. As indicated by the transcripts per million mapped reads (TPM) value, the number of full-length-*cdk6* transcripts (mRNA) in KB-C2 cells were 7-folds of that in KB-3-1 cells. The transcripts ENST00000265734 and ENST00000424848 corresponding to the full-length of CDK6 protein were indicated by *.
