# Supplementary material for: CDK6-PI3K signaling axis is an efficient target for attenuating ABCB1/P-gp mediated multi-drug resistance (MDR) in cancer cells
Source: Mol Cancer. 2022 Apr 22;21:103. doi: 10.1186/s12943-022-01524-w (PMC9027122; doi:10.1186/s12943-022-01524-w)
Supplement: Supplementary file 6 — Additional file 6: Table S1. Differential expression of CDK6, ABCB1, CDK4, PIK3CA, PIK3CB and GAPDH genes in KB-C2 and KB-C2-k.o.cdk6. Identical amounts of total RNA from KB-C2 and KB-C2-k.o.cdk6 cells were analyzed for transcriptome sequences and differential gene expression. The transcripts encoding full or effective length of proteins are listed. [file 12943_2022_1524_MOESM6_ESM.docx]

**Table S1.** Differential expression of CDK6, ABCB1, CDK4, PIK3CA, PIK3CB and GAPDH genes in KB-C2 and KB-C2-k.o.cdk6

**
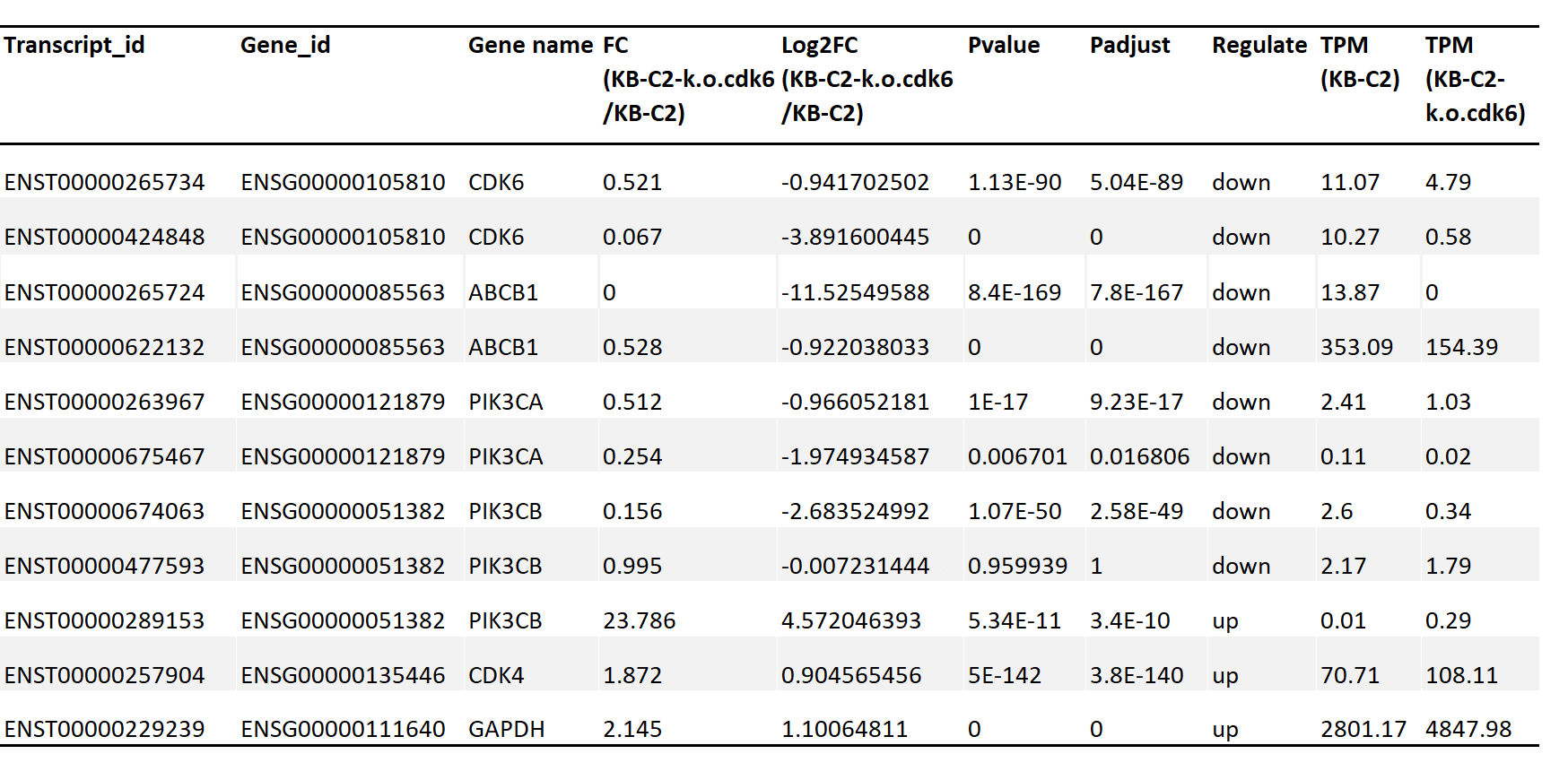
**

Identical amounts of total RNA from KB-C2 and KB-C2-k.o.cdk6 cells were analyzed for transcriptome sequences and differential gene expression. The transcripts encoding full or effective length of proteins are listed.
