# Supplementary material for: CDK6-PI3K signaling axis is an efficient target for attenuating ABCB1/P-gp mediated multi-drug resistance (MDR) in cancer cells
Source: Mol Cancer. 2022 Apr 22;21:103. doi: 10.1186/s12943-022-01524-w (PMC9027122; doi:10.1186/s12943-022-01524-w)

**Table S2.** Downregulation of the expression level of CDK6 in KB-C2-k.o.110α, KB-C2-k.o.110β, MX80-k.o.110α, or MX80-k.o.110β populations


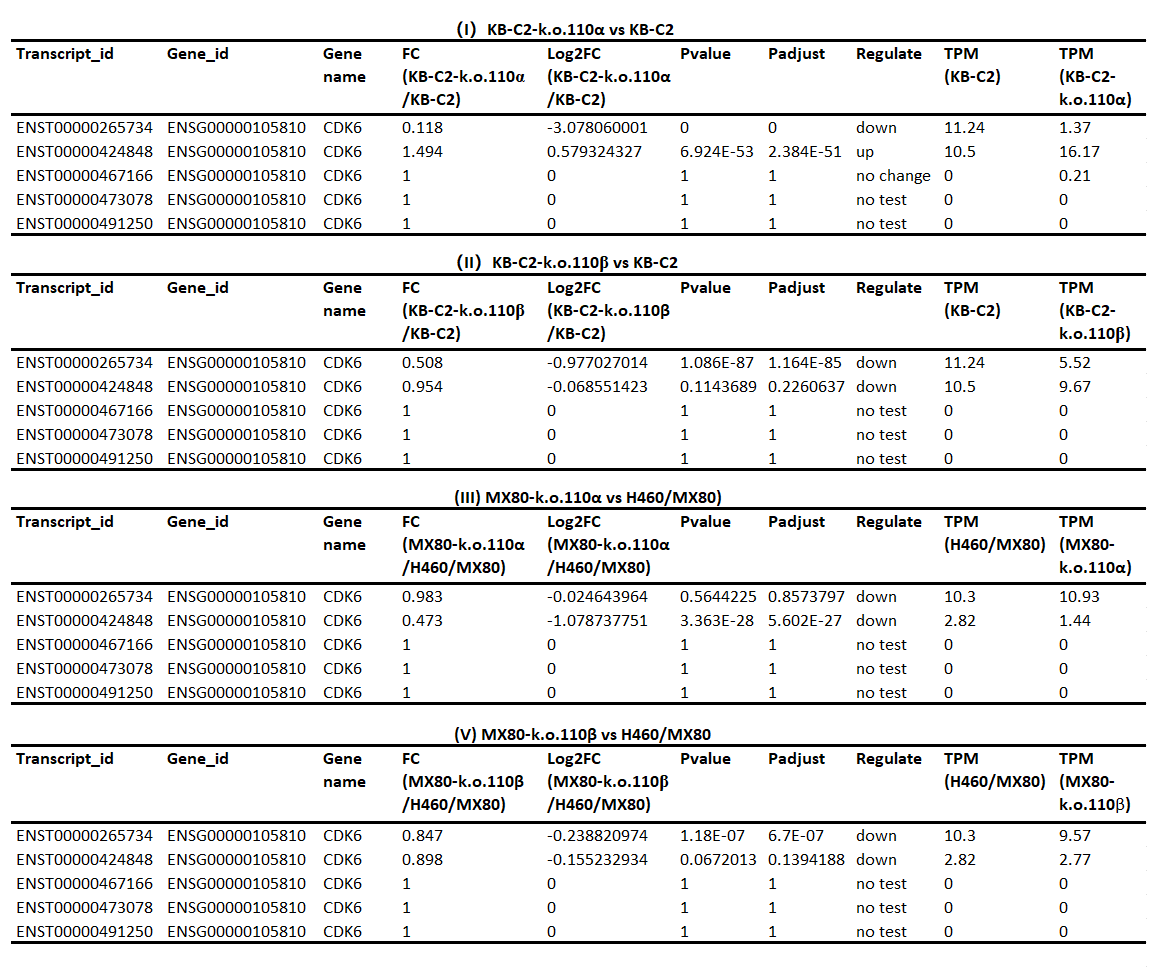

Supplement: Supplementary file 7 — Additional file 7: Table S2. Downregulation of the expression level of CDK6 in KB-C2-k.o.110α, KB-C2-k.o.110β, MX80-k.o.110α, or MX80-k.o.110β populations. [file 12943_2022_1524_MOESM7_ESM.docx]
