# Supplementary material for: CDK6-PI3K signaling axis is an efficient target for attenuating ABCB1/P-gp mediated multi-drug resistance (MDR) in cancer cells
Source: Mol Cancer. 2022 Apr 22;21:103. doi: 10.1186/s12943-022-01524-w (PMC9027122; doi:10.1186/s12943-022-01524-w)
Supplement: Supplementary file 8 — Additional file 8: Table S3. The percentages of non-apoptotic cells in the different phases of the cell cycle. The data were calculated based on the results in the histograms indicating a representative cell cycle (Fig. 6). [file 12943_2022_1524_MOESM8_ESM.docx]

**Table S3.** The percentages of non-apoptotic cells in the different phases of the cell cycle

|  | **Vehicle (0 µM colchicine)** | | |  | **Colchicine (1.25 µM)** | | |
| --- | --- | --- | --- | --- | --- | --- | --- |
|  | **KB-C2** | **KB-C2-k.o.cdk6** | **KB-C2-k.o.cdk4** |  | **KB-C2** | **KB-C2-k.o.cdk6** | **KB-C2-k.o.cdk4** |
| G0-G1 | 62.29 | 49.67 | 51.31 |  | 61.31 | 55.70 | 50.03 |
| S | 20.12 | 23.79 | 19.07 |  | 20.16 | 15.73 | 22.48 |
| G2-M | 17.59 | 26.54 | 29.63 |  | 18.53 | 28.58 | 27.49 |

The data were calculated based on the results in the histograms indicating a representative cell cycle (Fig. 6).
